# Small extracellular vesicles ameliorate peripheral neuropathy and enhance chemotherapy of oxaliplatin on ovarian cancer

**DOI:** 10.1002/jev2.12073

**Published:** 2021-03-04

**Authors:** Yi Zhang, Chao Li, Yi Qin, Pasquale Cepparulo, Michael Millman, Michael Chopp, Amy Kemper, Alexandra Szalad, Xuerong Lu, Lei Wang, Zheng Gang Zhang

**Affiliations:** ^1^ Department of Neurology Henry Ford Health System Detroit Michigan USA; ^2^ Department of Pathology Henry Ford Health System Detroit Michigan USA; ^3^ Department of Physics Oakland University Rochester Michigan USA

**Keywords:** Chemotherapy‐induced peripheral neuropathy, microRNAs, ovarian cancer, small extracellular vesicles

## Abstract

There are no effective treatments for chemotherapy induced peripheral neuropathy (CIPN). Small extracellular vesicles (sEVs) facilitate intercellular communication and mediate nerve function and tumour progression. We found that the treatment of mice bearing ovarian tumour with sEVs derived from cerebral endothelial cells (CEC‐sEVs) in combination with a chemo‐drug, oxaliplatin, robustly reduced oxaliplatin‐induced CIPN by decreasing oxaliplatin‐damaged myelination and nerve fibres of the sciatic nerve and significantly amplified chemotherapy of oxaliplatin by reducing tumour size. The combination therapy substantially increased a set of sEV cargo‐enriched miRNAs, but significantly reduced oxaliplatin‐increased proteins in the sciatic nerve and tumour tissues. Bioinformatics analysis revealed the altered miRNAs and proteins formed two distinct networks that regulate neuropathy and tumour growth, respectively. Intravenously administered CEC‐sEVs were internalized by axons of the sciatic nerve and cancer cells. Reduction of CEC‐sEV cargo miRNAs abolished the effects of CEC‐sEVs on oxaliplatin‐inhibited axonal growth and on amplification of the anti‐cancer effect in ovarian cancer cells, suggesting that alterations in the networks of miRNAs and proteins in recipient cells contribute to the therapeutic effect of CEC‐sEVs on CIPN. Together, the present study demonstrates that CEC‐sEVs suppressed CIPN and enhanced chemotherapy of oxaliplatin in the mouse bearing ovarian tumour.

## 1 INTRODUCTION

Chemotherapy‐induced peripheral neuropathy (CIPN) is one of the most common adverse complications of chemotherapy (Addington & Freimer, 2016; Argyriou et al., 2008; Kerckhove et al., 2017; Mcwhinney et al., 2009; Sisignano et al., 2014; Staff et al., 2017). Platinum‐based drugs are commonly used to treat ovarian, colorectal and lung cancers, while more than 70% of patients receiving oxaliplatin have neuropathy (Addington & Freimer, 2016; Argyriou et al., 2008; Kerckhove et al., 2017; Mcwhinney et al., 2009; Oun et al., 2018; Sisignano et al., 2014; Staff et al., 2017). The induced neurotoxicity often leads to platinum drug dose reductions, compromising efficiency of platinum drugs to suppress tumour progression. For cancer survivors, the CIPN symptoms can significantly impact quality of life (Addington & Freimer, 2016; Argyriou et al., 2008; Mcwhinney et al., 2009; Staff et al., 2017). The underlying cause of CIPN remains unknown. Platinum drugs cross‐link to DNA, forming the DNA/platinum adducts, and the amount of DNA crosslinks in dorsal root ganglia (DRG) neurons is correlated with the degree of neurotoxicity (Addington & Freimer, 2016; Argyriou et al., 2008; Mcwhinney et al., 2009; Sisignano et al., 2014). Other mechanisms include the rapid chelation of calcium by oxaliplatin‐induced oxalate and decreased cellular metabolism and axoplasmatic transport (Sisignano et al., 2014). Studies to develop a neuroprotective agent targeting these mechanisms have, to date, been unsuccessful in reducing CIPN (Addington & Freimer, 2016; Leal et al., 2014; Majithia et al., 2016 Mcwhinney et al., 2009; Sisignano et al., 2014). There is an imperative need to develop new therapies to CIPN. Challenges to develop such therapies include that a therapy should effectively inhibit CIPN, but not reduce antitumor efficacy.

**STATEMENT OF SIGNIFICANCE**

There are no effective therapeutic interventions for the treatment of chemotherapy‐induced peripheral neuropathy. We provide the first evidence that small extracellular vesicles derived from cerebral endothelial cells are effective to ameliorate platinum druginduced peripheral neuropathy and to sensitize the anti‐tumor effect of platinum drugs.

Axons of DRG neurons traverse a long distance to provide sensation to the toe and finger from their cell bodies that lie within or immediately adjacent to the spinal cord (Crispino et al., 2014; Jung et al., 2012). CIPN mainly affects sensory neurons in particular DRG neurons that are localized outside of the blood nerve barrier (Addington & Freimer, 2016; Argyriou et al., 2008; Feldman et al., 2017; Kerckhove et al., 2017; Mcwhinney et al., 2009; Staff et al., 2017). Platinum‐induced symptoms of peripheral neuropathy start from distal nerves of DRG neurons as a ‘glove and stocking’ sensory loss (Addington & Freimer, 2016; Argyriou et al., 2008; Mcwhinney et al., 2009). However, the majority of studies in CIPN have mainly analyzed the effect of platinum on cell bodies of DRG neurons (Cata et al., 2006; Wang et al., 2000). Emerging data indicate that distal axons are enriched with mRNAs, miRNAs and proteins, which provide effective ways for distal axons to respond to extrinsic signals and thereby communicate with their parent cell body (Crispino et al., 2014; Jung et al., 2012; Zhang et al., 2013). For example, alteration of the miRNA levels in distal axons of the cultured neurons promote axonal growth by locally suppressing miRNA targeting genes that inhibit axonal growth (Jia et al., 2018; Strickland et al., 2011).

Exosomes, the major constituents of small extracellular vesicles (sEVs, < 100 nm), are endosomal origin membranous nanovesicles (Meldolesi, 2018; Samanta et al., 2018; Théry et al., 2002; Van Niel et al., 2018; Witwer & Théry, 2019). EVs mediate intercellular communication by transferring cargo proteins, lipids, and genomic materials including miRNAs between source and recipient cells (Marcus & Leonard, 2013; Zhang & Chopp, 2016). EVs derived from healthy cells have been used for treatment of cancer and diabetic peripheral neuropathy. For example, sEVs derived from fibroblasts carrying siRNA against Kras have a therapeutic effect on tumour in a mouse model of pancreatic cancer (Kamerkar et al., 2017), while intravenous administration of sEVs derived from healthy Schwann cells remarkably ameliorate peripheral neuropathy in the mouse with diabetes (Wang et al., 2020). However, studies of the impact of exosomes on CIPN are limited. EV cargo share characteristics of their parent cells (Mathieu et al., 2019; Yáñez‐Mó et al., 2015). Our previous studies demonstrate that sEVs derived from mesenchymal stromal cells (MSC‐sEVs) contain miRNAs and proteins that mediate neuronal function (Zhang et al., 2017). MSC‐sEVs can be locally internalized by distal axons of cortical neurons and subsequently promote axonal growth even under axonal inhibitory conditions (Zhang et al., 2017). Here, we report that sEVs derived from cerebral endothelial cells (CEC‐sEVs) in combination with a platinum drug robustly suppress CIPN and enhance the anti‐tumour effect of oxaliplatin in nude mice bearing human ovarian cancer (OC). Intravenously administered CEC‐sEVs were internalized by nerve fibres of the sciatic nerve and cancer cells, and altered miRNA/protein networks that reduce neurotoxicity and amplify platinum drugs to kill cancer cells in the peripheral nerve and tumour, respectively.

## 2 RESULTS

### 2.1 CEC‐sEVs suppress oxaliplatin‐inhibited axonal growth of DRG neurons in vitro

**FIGURE 1:**
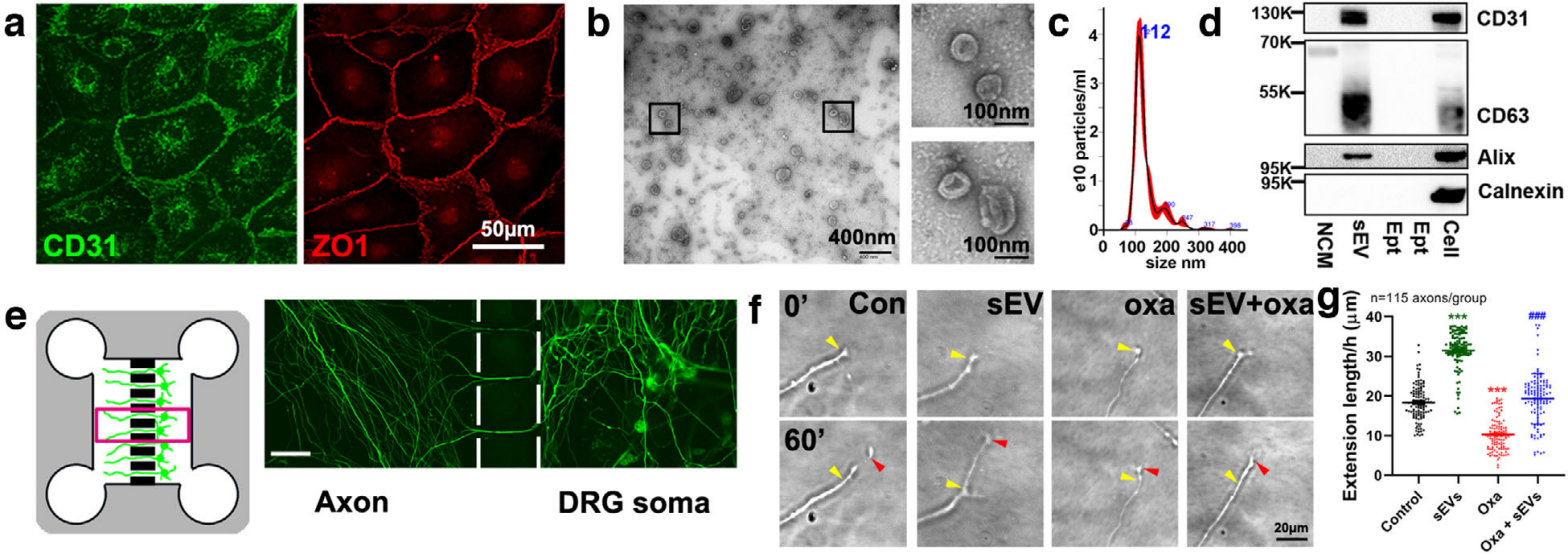
CEC‐sEVs promote axonal growth of DRG neurons with the presence of oxaliplatin. [Representative confocal microscopic images (a) show CECs are CD31 (green, CD31) and ZO1 positive (red, ZO‐1). Characterization of CEC‐sEVs by TEM (b), NTA (c) and Western blots (d), respectively. Schematic figure of the standard microfluidic device (SND150) along with an immunofluorescent image captured in the box area (e) shows DRG neurons grown in the cell body compartment (DRG soma) and their axons in 150 μm long microgrooves and in the axonal compartment (Axon). Representative time‐lapse microscopic images (f) of growth cone extension within 60 min and corresponding quantitative data of growth cone extension during a 24 h period (g), respectively, under control (con), CEC‐sEVs (sEV), oxaliplatin (oxa) and CEC‐sEVs in combination with oxaliplatin (sEV+oxa) conditions. Yellow and red arrows in panel G indicate the start (0′) and end positions (60′), respectively. One‐way ANOVA with Tukey's multiple comparisons test was used. *** *P* < 0.001 vs. control. N indicates the number of axonal growth cones. In panel D, NCM = the particles isolated from non‐conditioned medium, sEVs = CEC‐sEVs, Ept = the intentionally empty lanes, Cell = CEC lysate, K = the molecular weight Kda. Error bars indicate the standard error of the mean (SEM)]

A microfluidic device permits distal axons to grow into the axonal compartment from their parent cell bodies localized to the soma compartment (Figure 1e) (Zhang et al., 2013, 2016). To examine the direct effect of oxaliplatin on distal axons, oxaliplatin was applied to distal axons of embryonic DRG neurons cultured in the microfluidic device. Time‐lapse microscopic analysis showed that oxaliplatin inhibited axonal growth in a dose dependent manner with a half maximal inhibitory concentration, IC_50 _= 9.1 nM (Figure S1), indicating that oxaliplatin can directly act on axons to block axonal growth.

We then examined the effect of sEVs on axonal growth of DRG neurons. In a published study, we demonstrate that compared to MSC‐sEVs (Zhang et al., 2017), the CEC‐sEVs induce a more robust effect on promoting axonal growth of cortical neurons (Zhang et al., 2020). Therefore, we isolated and characterized EVs from primary human CECs (Figure 1a). The transmission electron microscopic (TEM) image showed doughnut‐shapes of collected EVs (Figure 1b) and nanoparticle tracking analysis (NTA) showed that the EVs had a mean diameter of 133±2.7 nm (Figure 1c). Western blot analysis showed that the EVs contained exosomal marker proteins, CD31, CD63 and Alix, whereas non‐conditioned media (NCM, Figure S2A) and whole CEC lysate (Figure 1d) did not have thee marker proteins. Additionally, calnexin, a protein expressed by CECs, was present in whole CEC lysate, but not in CEC‐sEVs (Figure 1d) (Théry et al., 2018). Collectively, these data indicate that EVs isolated from CEC conditioned medium are enriched with sEVs, consistent with the others’ results (Gardiner et al., 2016; Théry et al., 2006) and Minimal information for studies of extracellular vesicles 2018 (MISEV2018) report (Théry et al., 2018).

In pilot dose response experiments, we found that axonal application of CEC‐sEVs at doses of 3 × 10^7^, 3 × 10^8^ and 3 × 10^9^ particles/ml, but not a dose of 3 × 10^6^ particles/ml, significantly enhanced axonal growth of DRG neurons, while there were no significant differences among 3 × 10^7^ to 3 × 10^9^ particles/ml groups (Figure S3). Thus, a dose of 3 × 10^7^ particles/ml was selected for the in vitro experiments. Using a time‐lapse microscope, we found that application of CEC‐sEVs (3 × 10^7^ particles/ml) into the axonal compartment significantly promoted axonal growth (32 ± 2 μm/h vs. 18 ± 2 μm/h in the control group, *n* = 115, *P* < 0.001, Figure 1f,g). Moreover, compared with oxaliplatin alone, the application of CEC‐sEVs into the distal axons in the presence of oxaliplatin completely abolished oxaliplatin‐inhibited axonal growth (20 ± 2 μm/h vs. 10 ± 2 μm/h, *n* = 115, *P* < 0.001, Figure 1f,g). To assess the specificity of CEC‐sEVs on promoting axonal growth of DRG neurons, additional controls were employed including liposome mimics, NCM particles and EV‐depleted supernatant. Compared to PBS, liposome mimics, NCM particles and EV‐depleted supernatant did not significantly promote axonal growth (Figure S2B), suggesting that the effect of CEC‐sEVs on axonal growth is specific. These data indicate that CEC‐sEVs overcome oxaliplatin inhibited axonal growth.

### 2.2 CEC‐sEVs sensitize ovarian cancer cells to oxaliplatin in vitro

**FIGURE 2:**
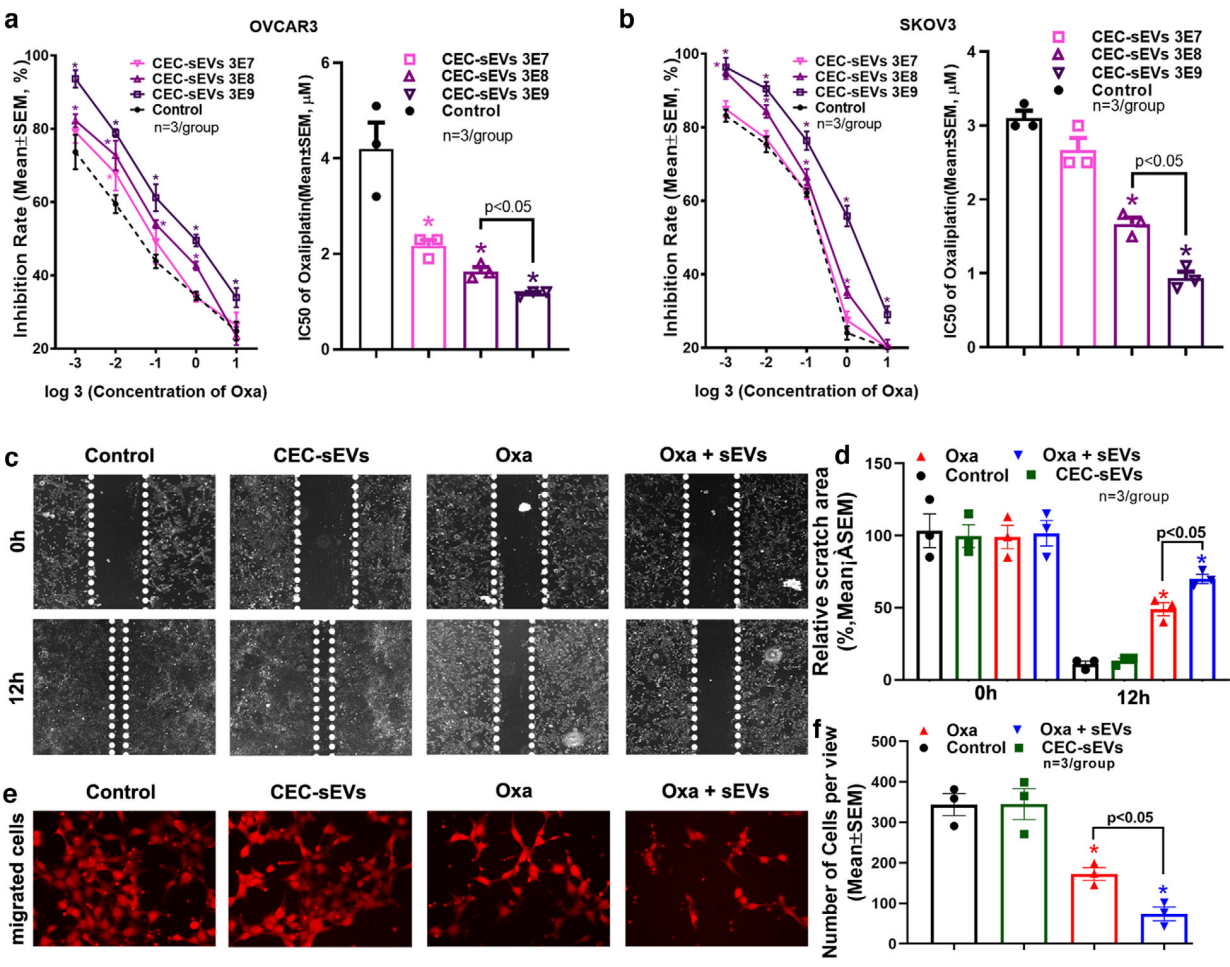
CEC‐sEVs enhanced anti‐cancer effects of oxaliplatin in OC cells. [Quantitative data of MTT cell viability assays on OVCAR3 (a) and SKOV3 (b) cells show the inhibition rates and corresponding IC_50_, respectively, of oxaliplatin in combination with different concentrations of CEC‐sEVs. Representative images (c) and quantitative data (d) show the results of a 12 h‐period wound healing assay of OVCAR3 cells treated with PBS (control), CEC‐sEVs, oxaliplatin (oxa) and CEC‐sEVs in combination with oxaliplatin (oxa + sEVs). Representative images (e) and quantitative data (f) show the results of the Transwell migration assay of OVCAR3 cells treated with different conditions for 24 h. N indicates the replications. One‐way ANOVA with Tukey's multiple comparisons test was used. * *P* < 0.05 vs. control; Error bars indicate the standard error of the mean (SEM)]

Next, we examined the effect of the combination of CEC‐sEVs and oxaliplatin on OC cells. Using two OC cell lines, SKOV3 and OVCAR3, we first assessed the effect of CEC‐sEVs on cancer cell viability. SKOV3 and OVCAR3 cell lines are derived from ascetic fluid and high grade serous ovarian carcinoma (HGSOC) of OC patients, respectively, and are widely used for pre‐clinical studies (Fogh et al., 1977; Mitra et al., 2015). The MTT assay showed that CEC‐sEVs at doses of 3 × 10^7^, 3 × 10^8^ and 3 × 10^9^ particles/ml did not significantly affect cancer cell viability. However, the combination of CEC‐sEVs at doses of 3 × 10^8^ and 3 × 10^9^ particles/ml, but not at a dose of 3 × 10^7^ particles/ml, with oxaliplatin significantly reduced viable cells and robustly decreased IC_50_s of oxaliplatin to these cancer cells compared with oxaliplatin alone (Figure 2ab). Using wound healing and transwell migration assays, we then examined the effect of combination treatment on OVCAR3 cell invasion. CEC‐sEVs at the dose of 3 × 10^8^ particles/ml in combination with oxaliplatin significantly blocked cancer cell migration compared with oxaliplatin alone, although CEC‐sEVs by themselves did not significantly affect OVCAR3 cell migration (Figure 2c‐f). In addition, the application of liposome mimics, NCM particles and EV‐depleted supernatant did not alter the IC_50_s of oxaliplatin to OVCAR3 and SKOV3 cells (Figure S2C), suggesting specificity of CEC‐sEVs on OC cells. Collectively, the in vitro data indicate that CEC‐sEVs sensitize the anti‐cancer effect of oxaliplatin on OC cell viability and invasion.

### 2.3 CEC‐sEVs mitigate oxaliplatin‐induced peripheral neuropathy and reduce tumour growth in tumour‐bearing mice

**FIGURE 3:**
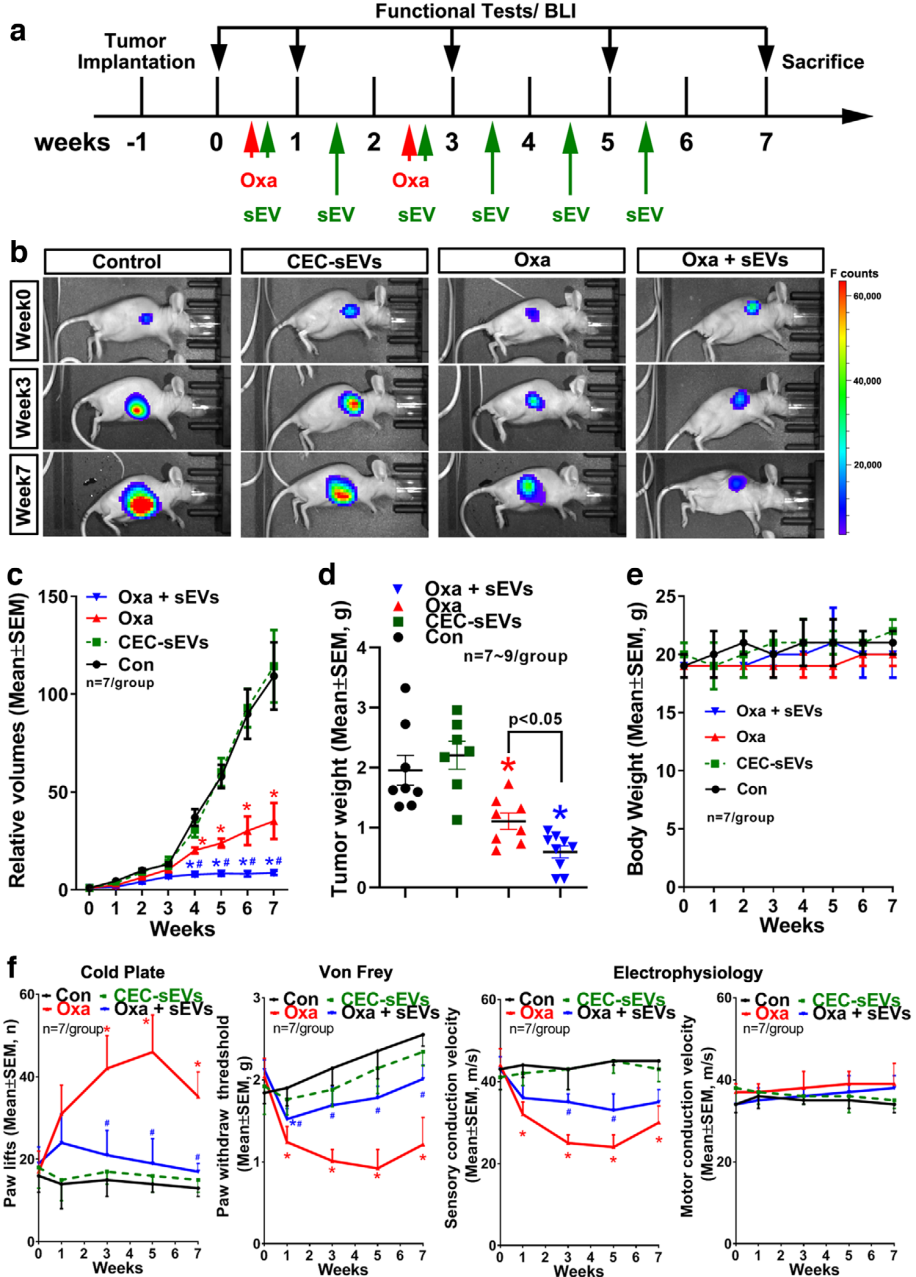
CEC‐sEVs enhance anti‐OC effects of oxaliplatin and ameliorate oxaliplatin‐induced peripheral neuropathy in nude mice bearing s.c. OC tumor. [A schematic (a) shows the experimental design. Representative images of luciferase signals acquired by BLI of nude mice bearing s.c. SKOV3/luc xenografts (b) and quantitative data of relative tumor volumes (c) and tumor weight (d) show the OC tumor progression in nude mice with different treatments, respecitively. The quantitative data show the longitudinally monitoring of animal body weight (e). The quantitative data (f) show the longitudinally monitoring of symptoms of peripheral neuropathy and nerve conduction velocities in sciatic nerves, respectively, of nude mice that received different treatments. N indicates the animal numbers in each group. One‐way ANOVA with Tukey's multiple comparisons test was used. * *P* < 0.05 vs. control; #, *P* < 0.05 vs. oxa. Error bars indicate the standard error of the mean (SEM)]

To examine whether the in vitro findings can be applied to animals, we generated a mouse model in which nude mice were subcutaneously (s.c.) xenografted with the human OC cell line, SKOV3/luc. When tumour development was confirmed 1 week after xenograft by non‐invasive imaging, the tumour‐bearing mice were treated with oxaliplatin (3.0 mg/kg, i.p.) daily for two rounds of five consecutive days per week at week 1 and week 3, with 1 week intervals between the treatments (Figure 3a), which mimics the clinical regimen (De Gramont et al., 2000). Longitudinal non‐invasive imaging analysis showed that oxaliplatin significantly reduced the tumour volumes by 45% and 68% at weeks 4 and 7, respectively, after the initial treatment (Figure 3b‐d). However, at week 1 after the first five doses of oxaliplatin, tumour‐bearing mice exhibited symptoms of cold hyperalgesia and tactile allodynia, which became worse at week 3 after completion of oxaliplatin treatment (Figure 3f). These mice also showed significant deficit of sensory conduction velocity (SCV), whereas motor conduction velocity (MCV) was not significantly affected (Figure 3f). The administration of oxaliplatin did not affect mouse body weight (Figure 3e), suggesting that the observed neuropathy is not likely induced by general toxicities of oxaliplatin.

We then examined the effects of CEC‐sEVs alone or in combination with oxaliplatin on tumour bearing mice. Based on preclinical studies in rodent, swine and primate, a dose of 3 × 10^11^ particles/injection was selected (Go et al., 2020; Wang et al., 2020; Williams et al., 2019; Xin et al., 2017). CEC‐sEVs (3 × 10^11^ particles/injection) were administered via a tail vein three times per week for six consecutive weeks, starting on the same day when oxaliplatin administration was initiated (Figure 3a). Compared with oxaliplatin alone, the combination of CEC‐sEVs with oxaliplatin significantly blocked oxaliplatin‐induced symptoms of cold hyperalgesia and tactile allodynia and impairment of SCV (Figure 3f). More importantly, the combination treatment significantly reduced tumour growth, although CEC‐sEVs alone did not significantly affect tumour growth (Figure 3b‐e).

**FIGURE 4:**
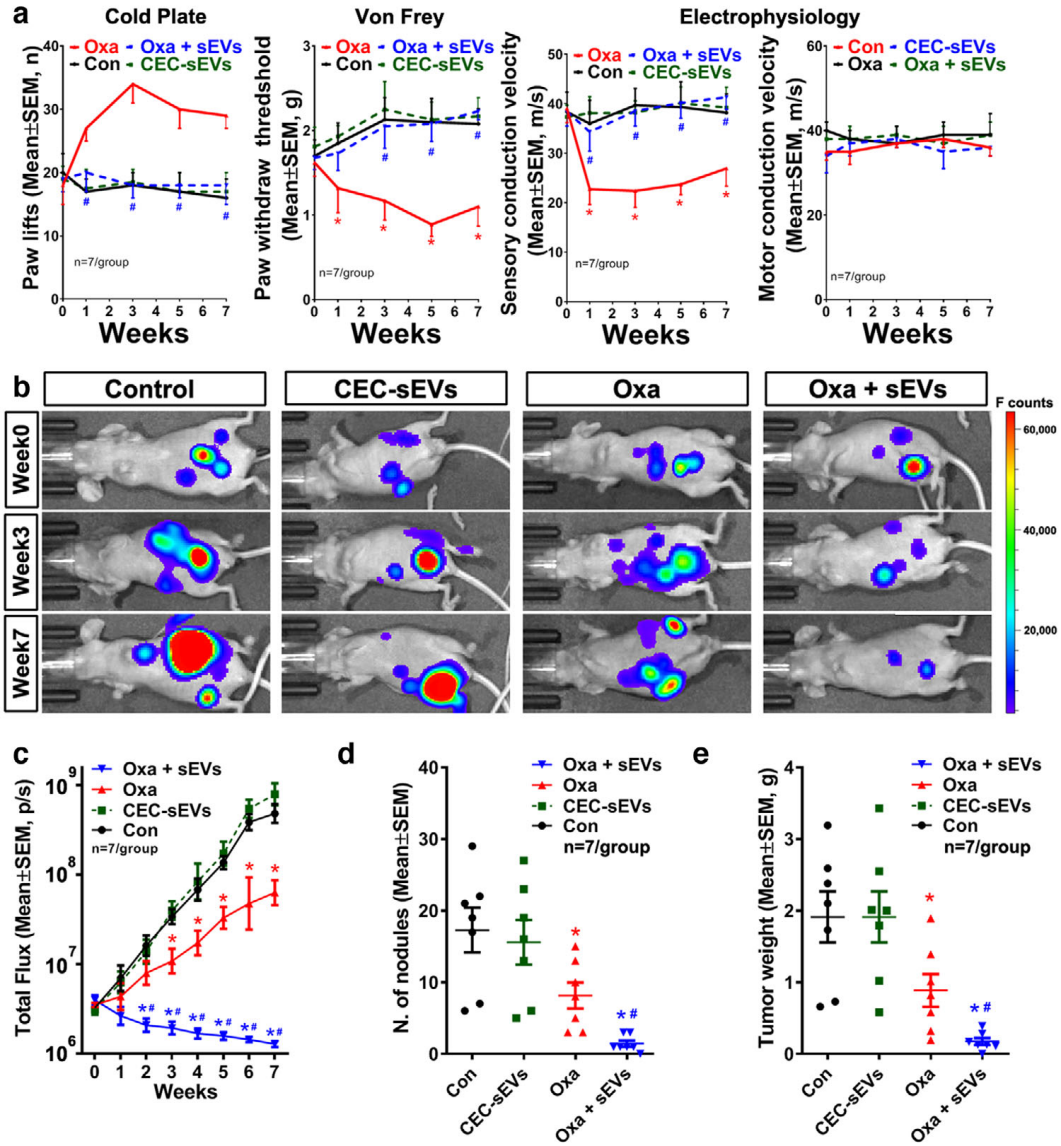
CEC‐sEVs enhanced anti‐OC effects of oxaliplatin and ameliorate oxaliplatin‐induced peripheral neuropathy in nude mice bearing i.p. OC tumor. [The quantitative data (a) show the longitudinally monitoring of symptoms of peripheral neuropathy and nerve conduction velocities in sciatic nerves, respectively, of nude mice bearing i.p. OVCAR3/luc xenografts and received different treatments. Representative ventral view images of luciferase signals acquired by BLI of nude mice (b) and quantitative data of relative tumor volume (c), total number of tumor nodules (d) and tumor weight (e) show the OC tumor progression in nude mice with different treatments. N indicates the animal numbers in each group. One‐way ANOVA with Tukey's multiple comparisons test was used. * *P* < 0.05 vs. control; #, *P* < 0.05 vs. oxa. Error bars indicate the standard error of the mean (SEM)]

To further verify the therapeutic effect of the combination treatment, we employed another mouse model of OC, in which nude mice were intraperitoneally xenografted with OVCAR3/luc cells. This model shows the peritoneal metastasis and the characteristics of human serous OC (Hu et al., 2005; Lengyel et al., 2014). Treatment of these tumour bearing mice with CEC‐sEVs in combination with oxaliplatin significantly reduced oxaliplatin‐induced peripheral neuropathy assayed by cold hyperalgesia, tactile allodynia and SCV (Figure 4a). The combination treatment also significantly decreased tumour growth and metastasis (Figure 4bc).

Collectively, these data indicate that CEC‐sEVs suppress oxaliplatin‐induced peripheral neuropathy and sensitize the anti‐tumour effect of oxaliplatin in tumour‐bearing mice.

### 2.4 CEC‐sEVs reduce oxaliplatin‐damaged axons and myelination

**FIGURE 5:**
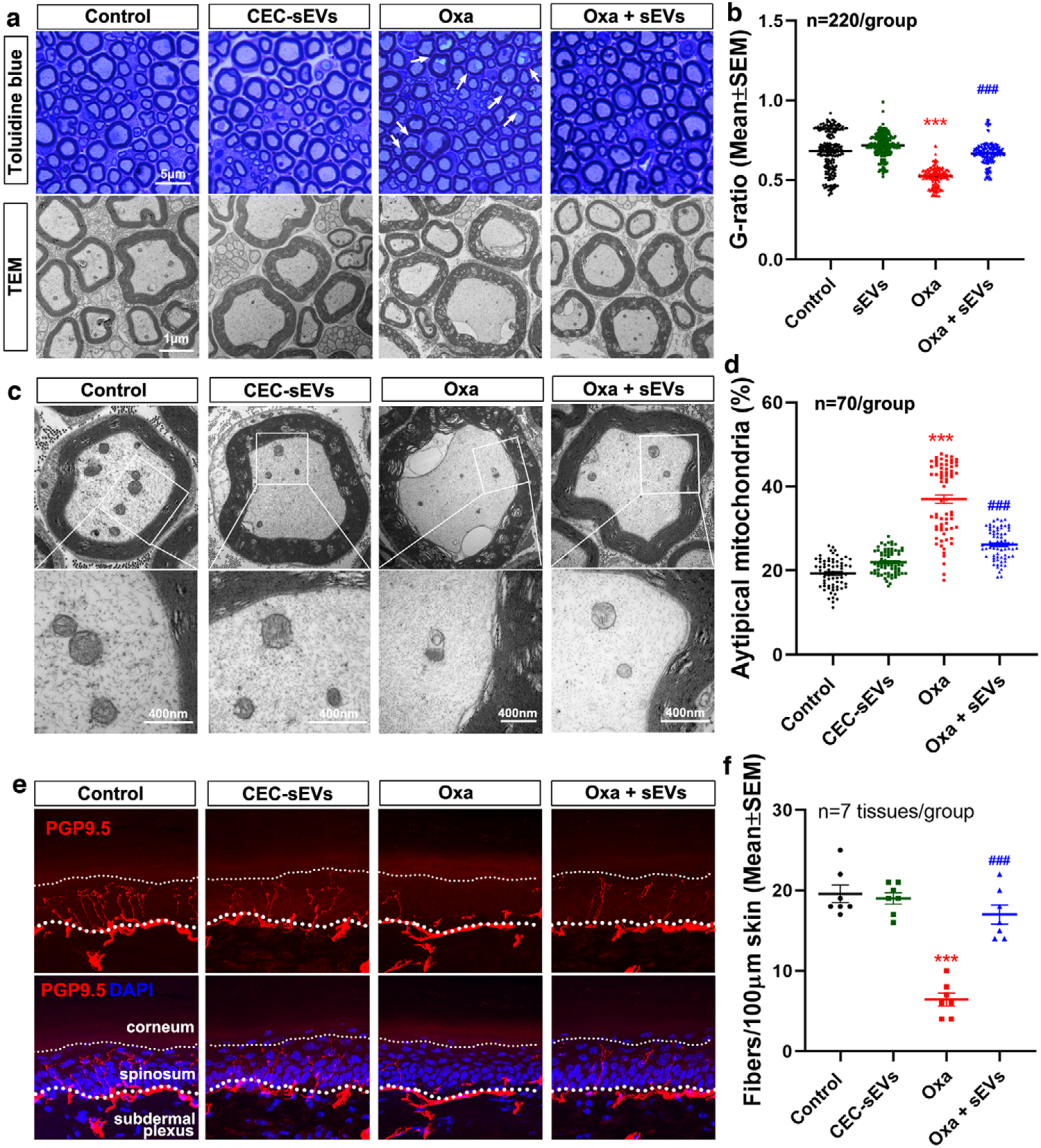
CEC‐sEVs reduce the damage to sciatic nerves and IENFs that are induced by oxaliplatin in nude mice. [Representative toluidine blue stain and TEM images (a) and the quantitative data of axon demyelination measured by G‐ratio (c) show the damage to axons in sciatic nerves of nude mice that received different treatments. The representative TEM images with enlarged areas of individual axons in sciatic nerves (b) and the quantitative data of vacuolated mitochondria (d) show the impairment of mitochondria in axons of sciatic nerves of nude mice that received different treatments. Representative confocal microscopic images and quantitative data of IENFs in footpad tissues of nude mice (e) show the lost of PGP9.5 positive IENFs (PGP9.5, red) in different groups. N in B and D indicates the number of axons were measured. One‐way ANOVA with Tukey's multiple comparisons test was used. *** *P* < 0.001 vs. control; ###, *P* < 0.001 vs. Oxa. Error bars indicate the standard error of the mean (SEM)]

**FIGURE 6:**
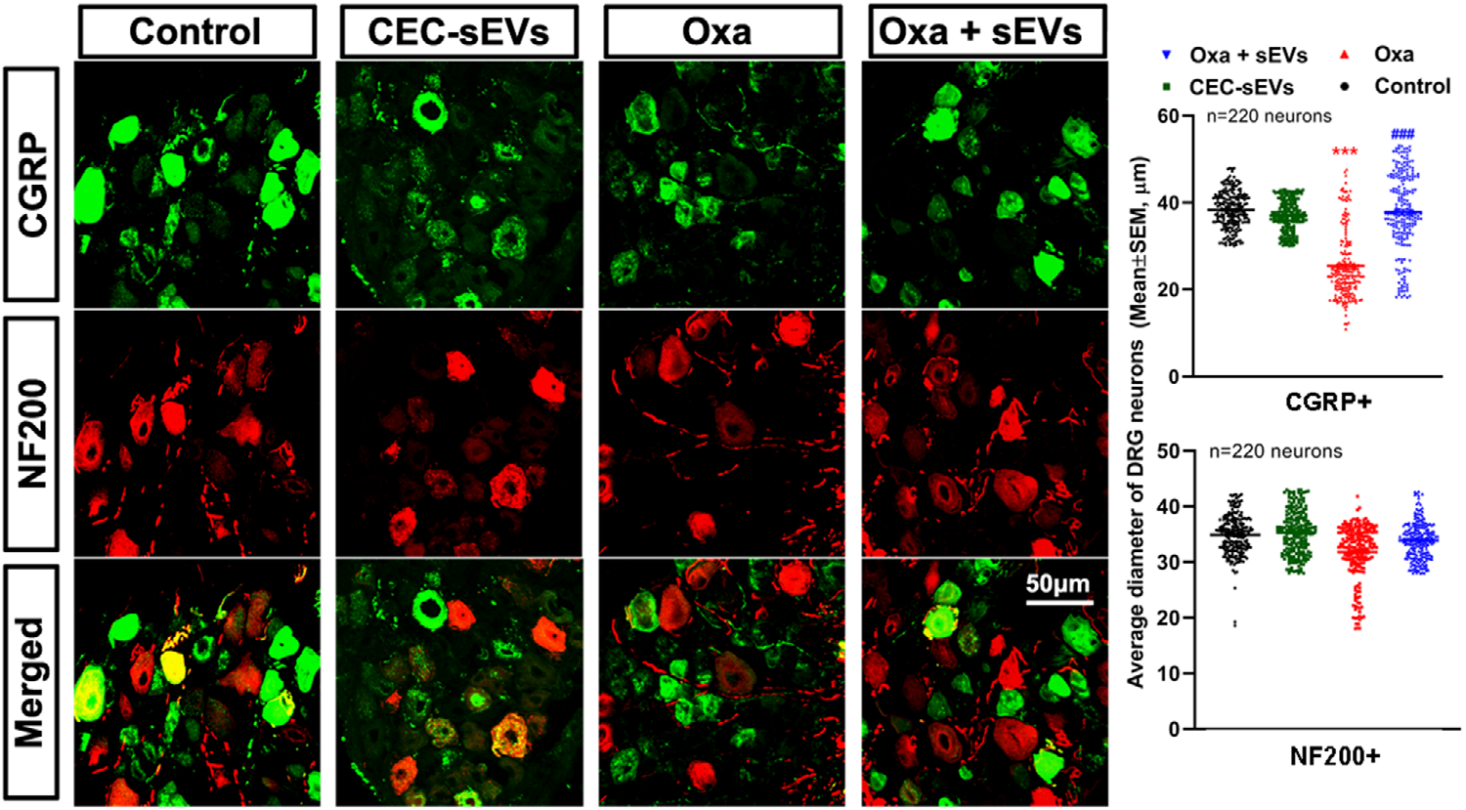
CEC‐sEVs reduce the damage to DRG neurons in nude mice induced by oxaliplatin. [Representative confocal microscopic images and quantitative data show the CGPR positive (CGRP, green) and NF200 positive (NF200, red) neurons in DRG tissue collected from nude mice that received different treatments. N indicates the number of DRG neurons were measured. One‐way ANOVA with Tukey's multiple comparisons test was used. *** *P* < 0.001 vs. control; ###, *P* < 0.001 vs. oxa. Error bars indicate the standard error of the mean (SEM)]

Histopathological analysis of sciatic nerve tissues acquired from the mice used in the aforementioned experiments revealed that oxaliplatin alone induced significant axonal shrinkage and demyelination assayed by myelin G‐ratio. TEM analysis showed that oxaliplatin induced axonal mitochondrial damage and myelin damage of the sciatic nerves (Figure 5a‐d). Immunostaining of dermal tissues showed that oxaliplatin significantly reduced the number of PGP9.5 positive intraepidermal nerve fibres (IENFs) in the hind plantar paw skin (Figure 5e,f). However, CEC‐sEVs significantly reduced oxaliplatin‐damaged axons, myelin, IENFs (Figure 5) and calcitonin gene‐related peptide (CGRP) positive DRG neurons (Figure 6). CEC‐sEVs in combination with oxaliplatin did not affect the number of neurofilament 200 (NF200) positive neurons (Figure 6). CGRP neurons are the primary afferent sensory neurons and chemotherapies specifically damage this neuron population (Mccoy et al., 2013; Palmiter, 2018; Zajączkowska et al., 2019). Monotherapy of CEC‐sEVs did not significantly affect DRG neurons, sciatic nerves and IEFS (Figures 5 and 6). These data suggest that increased nerve fibres and improved myelination by CEC‐sEVs likely contribute to the therapeutic effect of the combination treatment on CIPN.

### 2.5 CEC‐sEVs alter oxaliplatin‐induced miRNA and protein profiles in sciatic nerves and tumour cells

**FIGURE 7:**
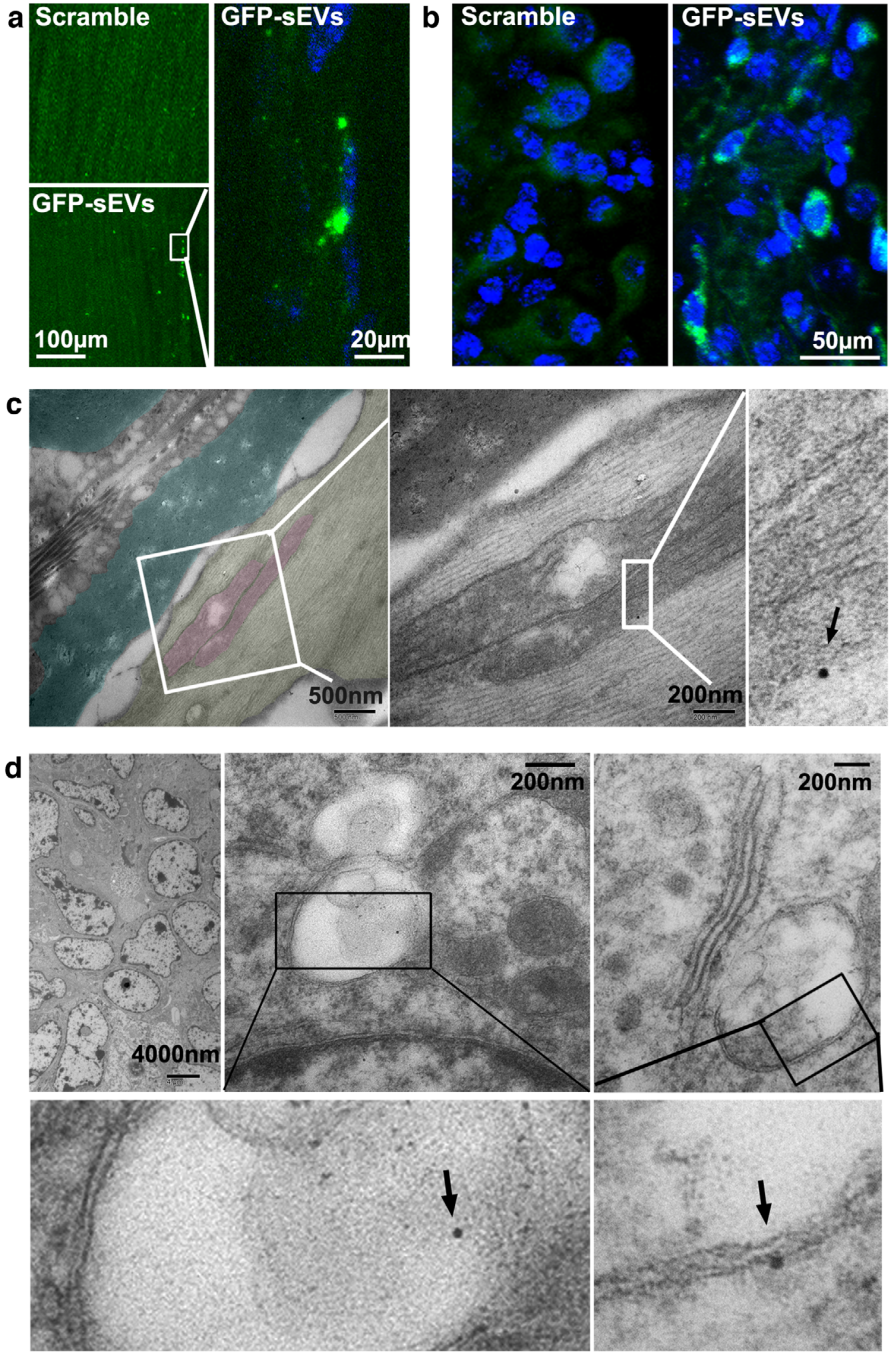
The internalization of CEC‐sEVs in sciatic nerves and OC tumor cells. [Confocal microscopic images show the presence of GFP signals in sciatic nerve fibres (a) and OC tumor cells (b) with the administration of GFP‐sEVs in nude mice bearing OC tumors. Representative TEM images with enlarged areas (c) show the GFP positive gold particle (black arrows) is associated with the mitochondria (pink) of damaged axons (yellow) with separated myelin sheath (dark green). Representative TEM images with enlarged areas (c) show the GFP positive gold particles (black arrows) are associated with MVBs in OC tumor cells]

Our published in vitro experiments demonstrate that MSC‐sEVs and CEC‐sEVs locally applied to the axon can be internalized by axons (Zhang et al., 2017, 2020). To examine whether intravenously administered CEC‐sEVs reach to sciatic nerves and tumours, CEC‐sEVs carrying CD63‐GFP (GFP‐sEVs) were intravenously administered to tumour‐bearing mice, and the mice were sacrificed 4 h after GFP‐sEVs administration. Confocal microscopic analysis showed the presence of strong green fluorescent signals in the nerve fibres and Schwann cells of sciatic nerve tissues (Figure 7a), and in the cytosol of tumour cells (Figure 7b). Moreover, TEM analysis showed the presence of immunogold GFP positive particles in axons of nerve fibres (Figure 7c) and in the cytoplasma of tumour cells (Figure 7d). Some of the immunogold positive particles were localized to the mitochondria in axons (Figure 7c, arrows) and later endosome of multivesicular bodies (MVB) in tumour cells (Figure 7d, arrows), respectively. However, there were no specific green fluorescent signals in the tissues acquired from mice treated with NCM particles (Figure S4). Collectively, these data provide evidence that the intravenously administered CEC‐sEVs are internalized by sciatic nerve fibres and ovarian cancer cells.

**FIGURE 8:**
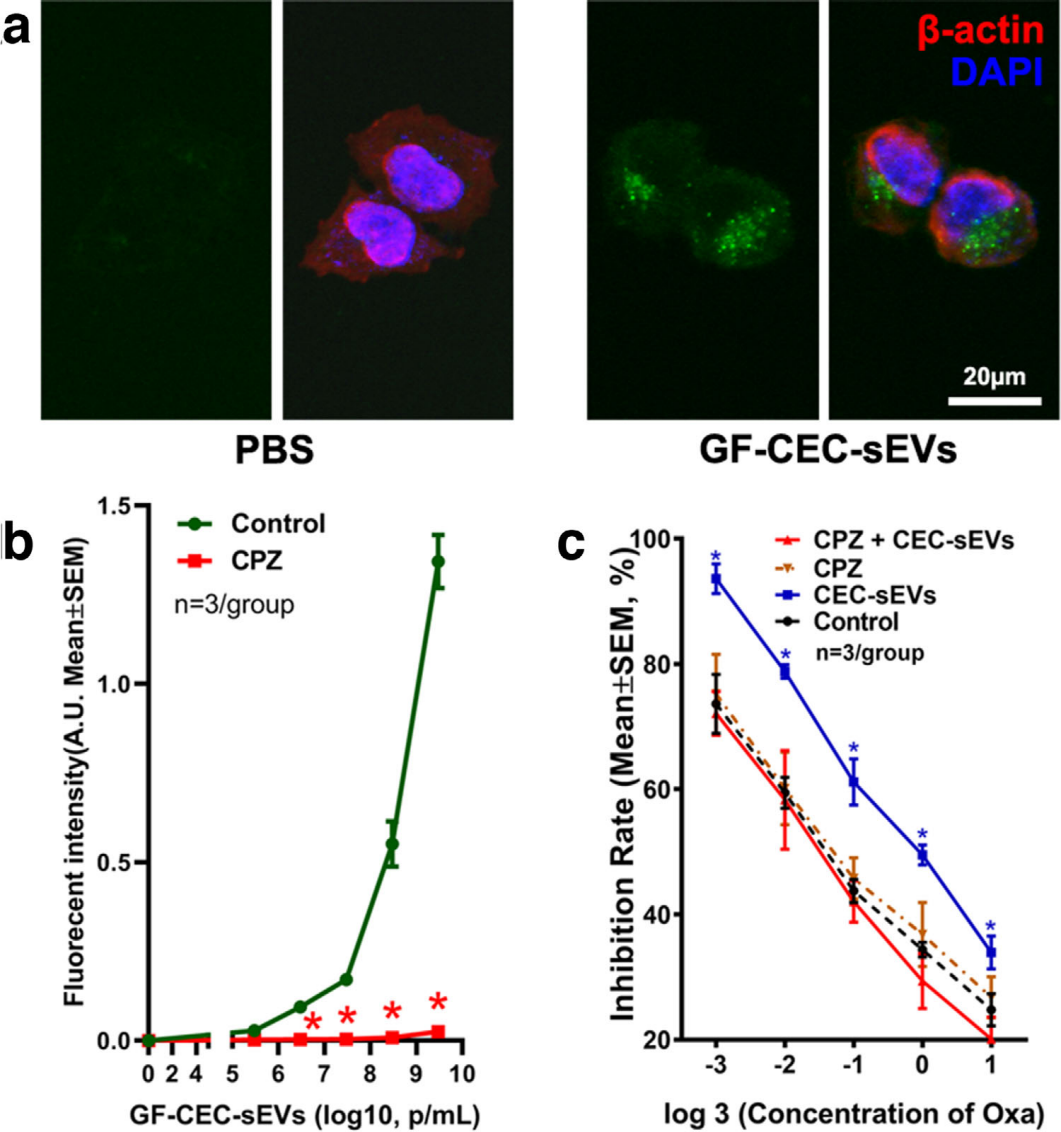
RNAs carried by CEC‐sEVs were internalized by OVCAR3 cells. [Representative confocal images (a) show the internalization of RNASelect Green Fluorescent labelled CEC‐sEVs (GF‐CEC‐sEVs) by OVCAR3 cells. Quantitative data of fluorescent intensity show that treatment of GF‐CEC‐sEVs dose‐dependently increased the green fluorescent signals in OVCAR3 cells (Control, b). The blockage of clathrin‐mediated sEV internalization by pre‐treatment of Chlorpromazine (5 μg/ml) abolished the increasing of green fluorescent signals in OVCAR3 cells (CPZ, b) and abolished the augmentation of oxaliplatin‐induced cell death by CEC‐sEVs (c). N indicates the number of replications. One‐way ANOVA with Tukey's multiple comparisons test was used. * *P* < 0.05 vs. control. Error bars indicate the standard error of the mean (SEM)]

Small EVs regulate recipient cell biology by delivering their cargo biomaterials upon internalization (Gonda et al., 2019; Mathieu et al., 2019; Shanmuganathan et al., 2018; Théry et al., 2002; Tkach & Théry, 2016). To examine whether cargo RNAs enter to recipient cells, we treated OVCAR3 cells with CEC‐sEVs carrying RNAs labelled by an RNASelect Green Fluorescent staining kit. Compared with the control, green fluorescent signals were dose‐dependently increased in OVCAR3 cells treated with GF‐CEC‐sEVs (Figure 8). In addition, incubation of OVCAR3 cells with GF‐CEC‐sEVs pre‐treated with Chlorpromazine abolished fluorescent signal augmentation in OVCAR3 cells and also eliminated CEC‐sEV‐enhanced oxaliplatin‐induced cell death (Figure 8). Chlorpromazine is a specific inhibitor of clathrin‐mediated endocytosis (Costa Verdera et al., 2017; Li et al., 2020; Tian et al., 2014). Collectively, these data suggest that CEC‐sEV cargo RNAs are internalized by OVCAR3 cells and that clathrin‐mediated endocytosis is involved in internalization of CEC‐sEVs by OVCAR3 cells.

**FIGURE 9:**
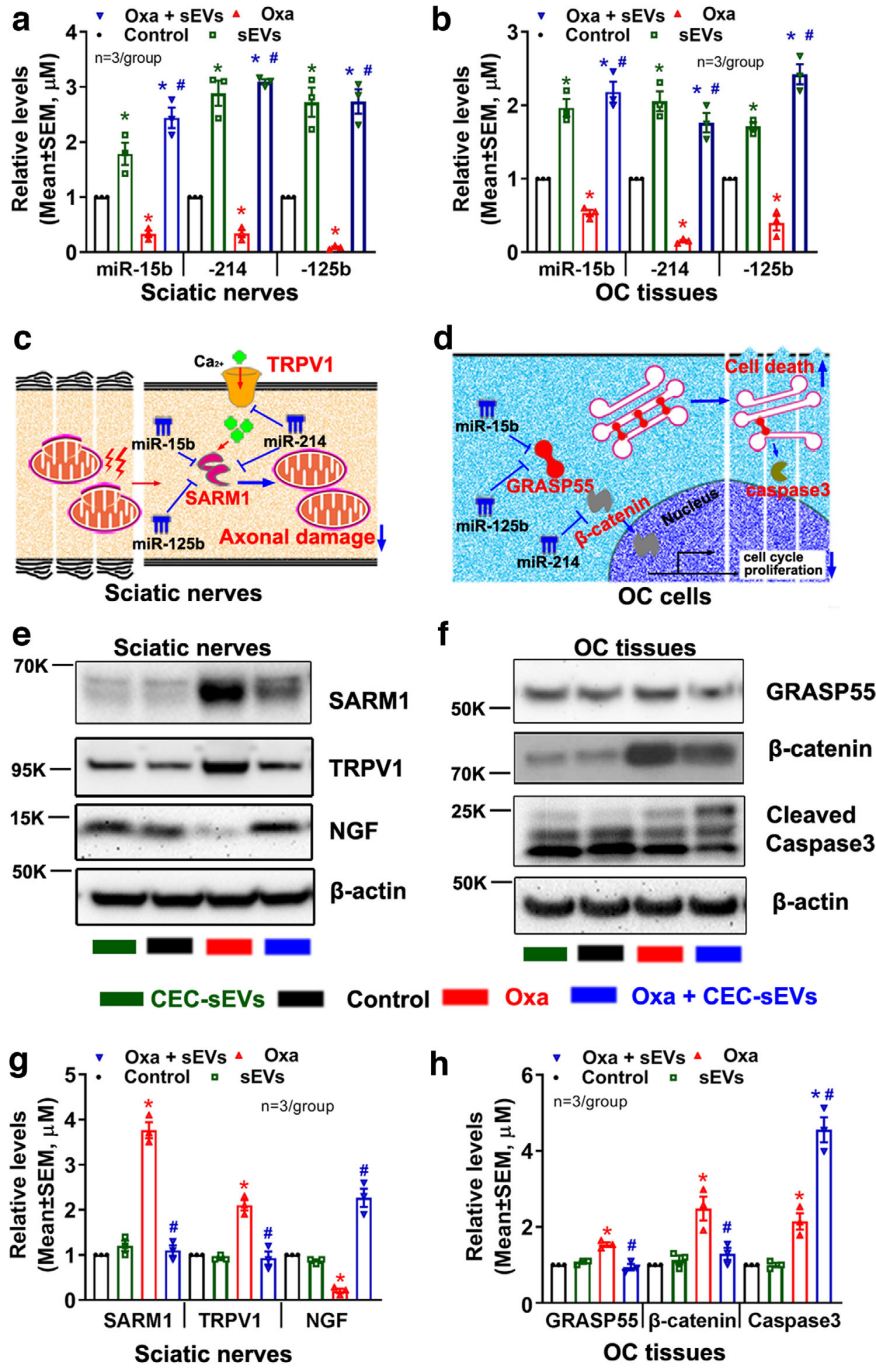
CEC‐sEVs alter oxaliplatin‐induced the changes of miRNAs and proteins in sciatic nerves and OC tumor. [qRT‐PCR results show the levels of miR‐15b, 214 and 125b in sciatic nerves (a) and OC tumor tissues (b), respectively, which were collected from nude mice that received different treatments. The schematic shows the miRNAs/target genes networks that contribute to the impairment of mitochondria in sciatic nerves (c) and OC cell apoptosis (d), respectively. Representative Western blot results and their quantitative data show the levels of proteins in sciatic nerves (e, g) and in OC tumor tissues (f, h), respectively. N indicates the number of replications. K indicates the molecular weight KDa. One‐way ANOVA with Tukey's multiple comparisons test was used. * *P* < 0.05 vs. control; #, *P* < 0.05 vs. oxa. Error bars indicate the standard error of the mean (SEM)]

To examine whether CEC‐sEV cargo miRNAs affect sciatic nerve fibres and tumour cells, we first examined the CEC‐sEV cargo miRNA profiles by means of miRNA‐seq and found that CEC‐sEVs contained 677 miRNAs (Supplemental Excel file). Among them, miR‐15b, ‐214 and ‐125b were enriched, as confirmed by qRT‐PCR (Table 1). In contrast, qRT‐PCR analysis of sciatic nerve and tumour tissues of tumour bearing mice showed that compared with non‐oxaliplatin treated tissues (control tissues), levels of miR‐15b, ‐214 and ‐125b in oxaliplatin‐treated sciatic nerve and tumour tissues were significantly reduced (Figure 9ab). CEC‐sEVs in combination with oxaliplatin significantly elevated these three miRNAs in sciatic nerve and tumour tissues (Figure 9ab). Bioinformatics analysis with Ingenuity Pathways Analysis (IPA) showed that miR‐15b, ‐214 and ‐125b along with their putative target genes formed at least two distinct networks that mediate neuronal function and tumour development, respectively (Figure 9c,d). Using Western blot, we thus examined protein levels of two target genes, Transient receptor potential cation channel subfamily V member 1 (TRPV1) and Sterile alpha and TIR motif containing 1 (Sarm1), in the sciatic nerve tissue, which are involved in CIPN (Geisler et al., 2016; Hara et al., 2013; Hohmann et al., 2017; Szallasi et al., 2007). Compared with the control sciatic nerve tissue, TRPV1 and SARM1 proteins were significantly elevated in oxaliplatin‐treated sciatic nerve tissue. However, protein levels of TRPV1 and SARM1 were significantly reduced in the sciatic nerve from mice treated with CEC‐sEVs in combination with oxaliplatin compared with protein levels from mice treated with oxaliplatin alone (Figure 9c,d). Although monotherapy of CEC‐sEVs significantly increased these three miRNAs, CEC‐sEVs alone did not significantly alter the levels of TRPV1 and SARM1 in sciatic nerve tissues compared to the control (Figure 9e‐h). Next, we examined protein levels of Golgi reassembly‐stacking protein of 55 kDa (GRASP55) and β‐catenin in the tumour tissue. Compared to the control tumour tissue, protein levels of GRASP55 and β‐catenin were significantly elevated in oxaliplatin‐treated tumour, whereas monotherapy of CEC‐sEVs did not affect these two protein levels (Figure 9fh). However, compared to oxaliplatin alone, CEC‐sEVs in combination oxaliplatin significantly reduced oxaliplatin‐augmented GRASP55 and β‐catenin (Figure 9fh). Collectively, these data suggest that interactions between CEC‐sEV enriched cargo miR‐15b, ‐214 and ‐125b and their target genes in recipient peripheral nerves and tumour cells are cell type dependent. An inverse relationship of the miRNAs and the proteins that mediate CIPN and tumour development suggest that these networks altered by CEC‐sEVs and oxaliplatin likely contribute to the improved neuropathy and suppressed tumour growth.

**TABLE 1:** Abundant miRNAs in CEC‐sEVs

miRNAs	Average CT	SD (*n* = 3)
miR‐15b‐5p	21.18	3.8
miR‐125b‐5p	22.99	4.3
miR‐214‐3p	24.02	2.7
miR‐21‐5p	27.79	4.3
miR‐100‐5p	27.89	5.1
let‐7i‐5p	36.45	4.2
miR‐151a‐3p	36.97	1.9
miR‐221‐3p	38.14	1.9
miR‐222‐3p	39.88	2.3
miR‐10b‐5p	>40	NA

Epithelial mesenchymal transition (EMT) mediates metastasis (Chen et al., 2017; Davidson et al., 2012; Fang et al., 2017). We found that OVCAR3 cells cultured under regular condition expressed an epithelial marker, E‐cadherin, but not a mesenchymal marker, Vimentin (Figure S5), which is consistent with other's results (Yi et al., 2015). When they were cultured in Matrigel, OVCAR3 cells expressed Vimentin and downregulated E‐cadherin (Figure S5). However, CEC‐sEVs in combination with oxaliplatin substantially reduced Vimentin protein (Figure S5) compared with monotherapy of oxaliplatin, whereas CEC‐sEVs alone did not reduce Vimentin protein (Figure S5). These data suggest that the combination of CEC‐sEVs with oxaliplatin reduces EMT. The DNA/platinum adducts induce cytotoxicity (Johnson et al., 1994; Wang et al., 2018). We thus examined the effect of CEC‐sEVs on oxaliplatin‐DNA interstrand crosslinks. The treatment of OVCAR3 cells with CEC‐sEVs at 3 × 10^8^ particles/ml did not significantly change the oxaliplatin‐DNA interstrand crosslinks in OVCAR3 cells (Table S1).

**FIGURE 10:**
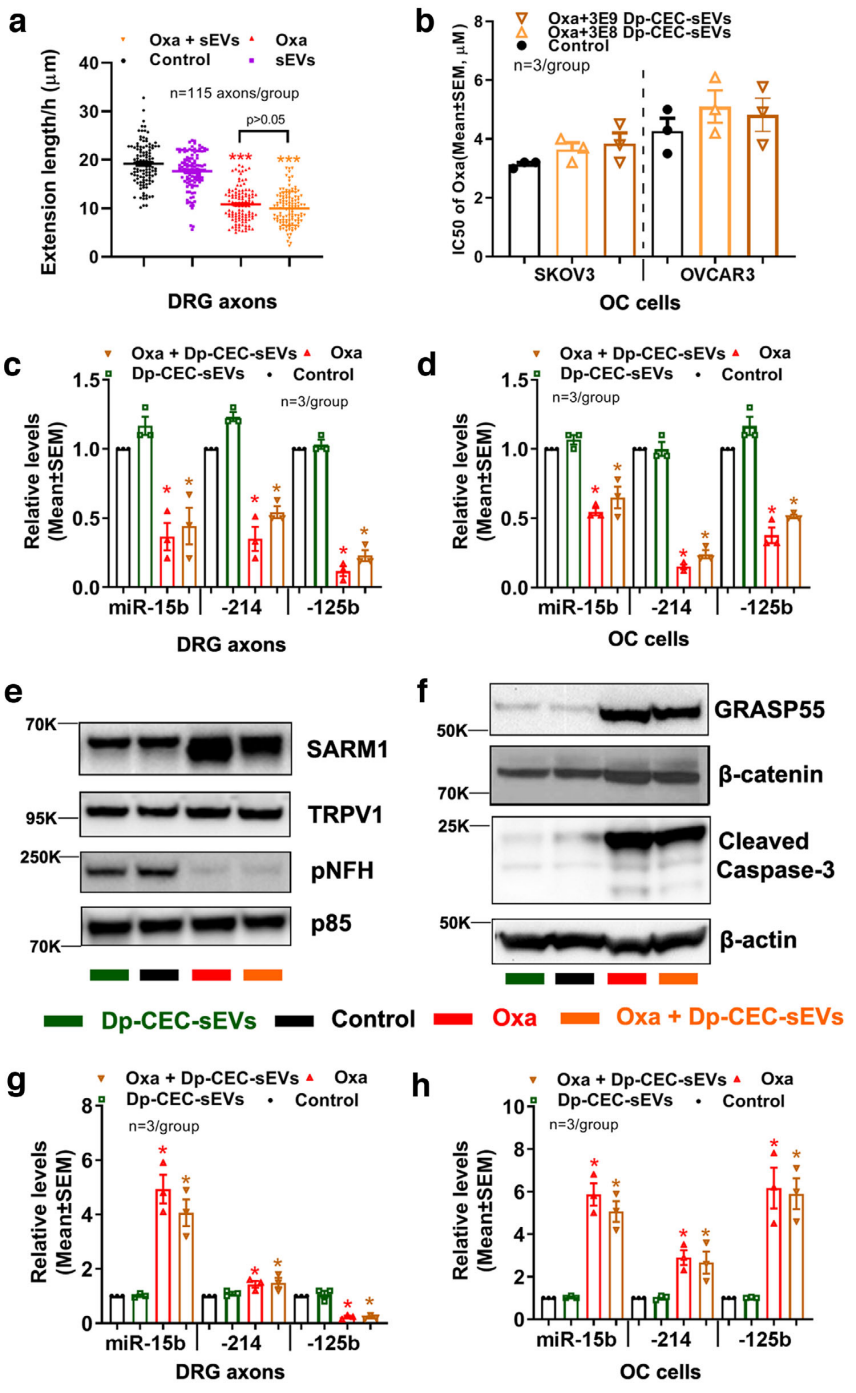
Dp‐CEC‐sEVs do not promote axonal growth and sensitize OC cells to oxaliplatin, and do not alter oxaliplatin‐induced the changes of miRNAs and proteins in axons and OC cells. [Quantitative data in a show growth cone extension of DRG axons within 60 min and treated with PBS (con), Oxaliplatin (oxa), Dp‐CEC‐sEVs and oxaliplatin in combination with Dp‐CEC‐sEVs (oxa+ Dp‐CEC‐sEVs). Quantitative data in b show IC_50_ of oxaliplatin in combination with different concentration of Dp‐CEC‐sEVs on SKOV3 and OVCAR3 cells, respectively. qRT‐PCR results show the levels of miR‐15b, 214 and 125b in axons of DRG neurons (c) and in SKOV3 cells (d), respectively, which were received different treatments. Representative Western blots results and their quantitative data show the levels of proteins in axons of DRGs (e, g) and in SKOV3 cells (f, h), respectively, which received different treatments. N in b‐d, g and h indicate the number of replications. K indicates the molecular weight KDa. One‐way ANOVA with Tukey's multiple comparisons test was used. * *P* < 0.05, *** *P* < 0.001 vs. control; #, *P* < 0.05 vs. oxa. Error bars indicate the standard error of the mean (SEM)]

To determine the effect of CEC‐sEV cargo miRNAs on axons and OC cells, we isolated sEVs from CECs transfected with shRNA against Dicer (Dp‐CEC‐sEVs), a key gene for miRNA biogenesis (Kim, 2005; Thomou et al., 2017). Quantitative RT‐PCR analysis showed reduction of Dicer‐related miRNAs in Dp‐CEC‐sEVs (Table 2). Treatment of axons of the DRG neurons or SKOV3 cells with Dp‐CEC‐sEVs did not significantly increase the miR‐15b, ‐214 and ‐125b levels in axons of DRG neurons and SKOV3 cells, did not overcome the oxaliplatin‐inhibited axonal growth of DRG neurons, and did not amplify the effect of anti‐cancer cell of oxaliplatin on SKOV3 cells, respectively, compared with oxaliplatin alone (Figure 10a to d). Western blot analysis showed that the treatment of Dp‐CEC‐sEVs did not significantly alter oxaliplatin‐augmented TRPV1 and SARM1 in DRG axons (Figure 10eg) and oxaliplatin‐increased GRASP55 and β‐catenin in SKOV3 cells (Figure 10fh), respectively. Compared to the control, monotherapy of Dp‐CEC‐sEVs did not affect the levels of TRPV1 and SARM1 in DRG axons (Figure 10eg) and GRASP55 and β‐catenin in SKOV3 cells (Figure 10fh), respectively. The data indicate that cargo miRNAs are required for the therapeutic effects of CEC‐sEVs on DRG axons and OC cells.

**TABLE 2:** miRNAs in Dp‐CEC‐sEVs

miRNAs	Average CT	SD (*n* = 3)
miR‐15b‐5p	36.23	1.6
miR‐125b‐5p	37.38	1.5
miR‐214‐3p	37.09	1.7
miR‐21‐5p	35.12	1.0
miR‐100‐5p	38.40	2.1
let‐7i‐5p	39.15	3.2
miR‐151a‐3p	35.13	1.7
miR‐221‐3p	39.94	2.1
miR‐222‐3p	>40	NA
miR‐10b‐5p	>40	NA

**FIGURE 11:**
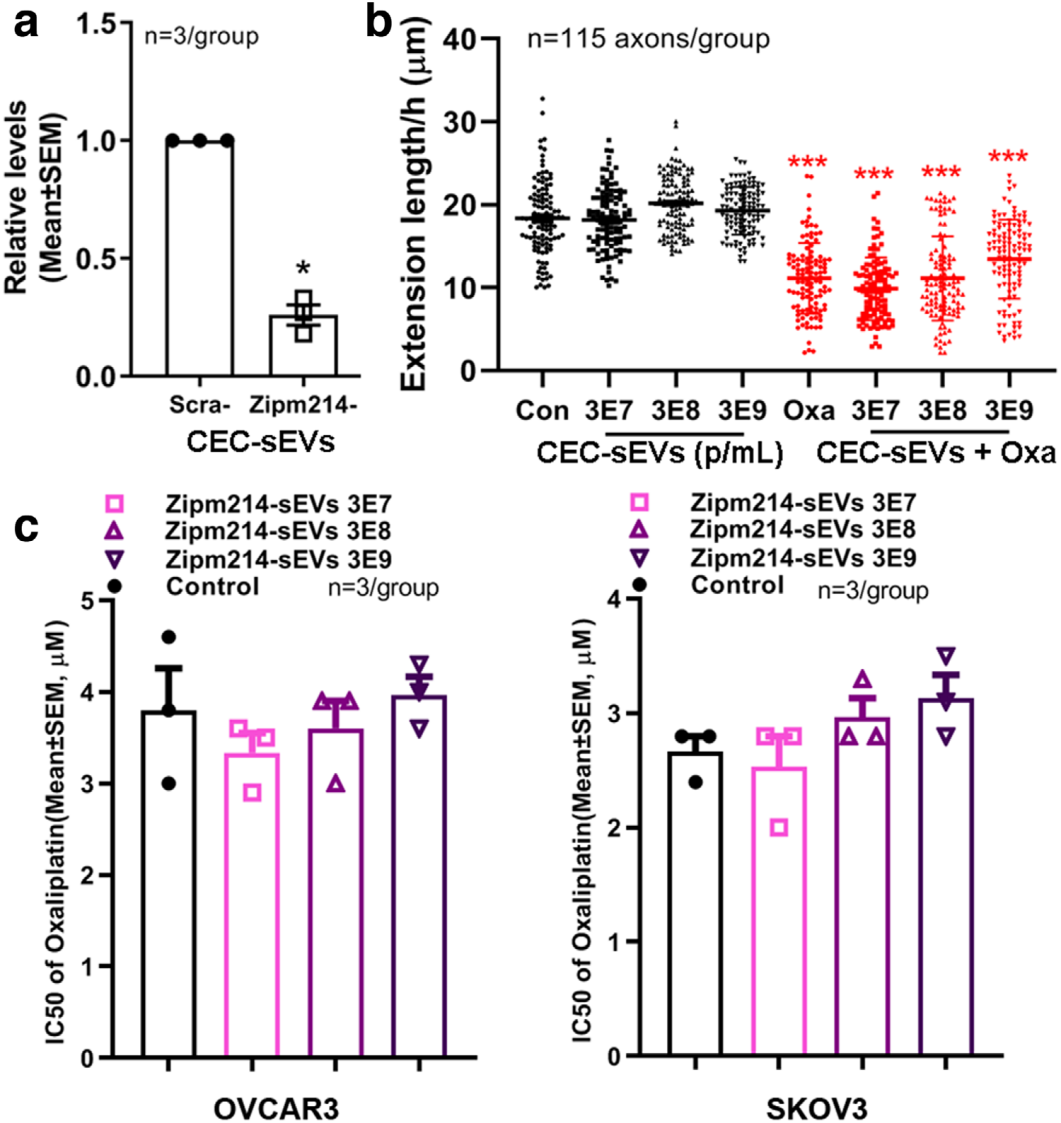
CEC‐sEVs carrying reduced miR‐214 substantially attenuate the effects of CEC‐sEVs on DRG axons and OC cells. [Quantitative RT‐PCR data (a) show that the levels of miR‐214‐3p were significantly decreased in CEC‐sEVs isolated from CECs transfected with lentiviral vector carrying shRNA against miR‐214 (Zipm214‐CEC‐sEVs) compared with CEC‐sEVs isolated from CECs transfected a scramble control vector (scra‐CEC‐sEVs). Quantitative data (b) show the effects of different concentration of Zipm214‐CEC‐sEVs (Zip‐sEVs) on growth cone extension of DRG neurons during a 24 h period (b) with or without oxaliplatin (9.1 nM). Quantitative data of MTT assay (c) show the effects of different concentration of Zipm214‐CEC‐sEVs on cell viability of OVCAR3 and SKOV3 cells under IC50 of oxaliplatin. N indicates the number of replications. One‐way ANOVA with Tukey's multiple comparisons test was used. * *P* < 0.05, *** *P* < 0.001 vs. control. Error bars indicate the standard error of the mean (SEM)]

Ablation of Dicer likely affects many Dicer‐related miRNAs (Gordillo et al., 2014; Hancock et al., 2014; Suárez et al., 2007). As proof of principle, we then specifically examined one of the three miRNAs, miR‐214. Small EVs (Zipm214‐CEC‐sEVs) were isolated from the supernatant of CECs transfected by a lentiviral vector carrying shRNA against miR‐214 (MZIP214‐PA‐1, SystemBio). Quantitative RT‐PCR analysis revealed that levels of miRNA‐214 within Zipm214‐CEC‐sEVs were 74% lower than sEVs (scr‐CEC‐sEVs) from CECs transfected with a control vector (MZIP000‐PA‐1, SystemBio) (Figure 11a). The Zipm214‐CEC‐sEVs did not overcome the inhibitory effect of oxaliplatin on axonal growth of DRG neurons and did not enhance the anti‐tumour effect of oxaliplatin on OVCAR3 and SKOV3 cells compared with scr‐CEC‐sEVs (Figure 11bc). Sarm1 gene is directly suppressed by miR‐214 (Figure S6). β‐catenin is validated genes targeted by miR‐214 (Liu et al., 2018; Xia et al., 2012). We thus overexpressed Sarm1 in DRG neurons and activated β‐catenin in OVCAR3 cells by Wnt 3a, respectively. The application of CEC‐sEVs to Sarm1 overexpressed DRG neurons did not significantly enhance the axonal growth (Figure S7). In OVCAR3 cells, the pre‐treatment of OVCAR3 cells with Wnt3a significantly increased the levels of β‐catenin and abolished chemosensitization effects of CEC‐sEVs on these cells (Figure S7). Collectively, these data suggest that the enriched miR‐214 in CEC‐sEVs could act on their target genes in recipient cells, leading to changes of recipient cell function.

## 3 DISCUSSION

The present study demonstrated that CEC‐sEVs in combination with oxaliplatin robustly reduced oxaliplatin‐induced peripheral neuropathy and amplified the anti‐tumour effect of oxaliplatin in the mouse bearing ovarian tumour. CEC‐sEVs intravenously administered were internalized by axons of the sciatic nerve and cancer cells. CEC‐sEVs in combination with oxaliplatin substantially increased a set of sEV cargo‐enriched miRNAs in sciatic nerve and tumour tissues, and significantly reduced oxaliplatin‐increased proteins in the sciatic nerve and tumour tissues. Reduction of CEC‐sEV cargo miRNAs abolished the effect of CEC‐sEVs on oxaliplatin‐inhibited axonal growth of DRG neurons and on oxaliplatin‐enhanced anti‐ ovarian cancer cells. Together, we provide evidence that CEC‐sEVs suppress oxaliplatin‐induced peripheral neuropathy and sensitize anti‐tumour effect of oxaliplatin in the mouse bearing ovarian tumour. The present study also suggests a potential mechanism by which alterations in the networks of miRNAs and proteins in recipient cells contribute to the therapeutic effect of CEC‐sEVs on CIPN.

Although agents targeting neuroprotection (Cascinu et al., 2002; Chen et al., 2017; Weber et al., 2013), anti‐inflammation (Mao‐Ying et al., 2014; Pachman et al., 2017) and anti‐oxidation (Carvalho et al., 2017; Hilpert et al., 2005; Yri et al., 2009) have shown promising effects on the prevention and treatment of CIPN in patients and in animal models, challenges remain to develop therapies for CIPN in which a therapy effectively inhibits CIPN, but does not reduce antitumor efficacy. Platinum‐induced symptoms of peripheral neuropathy start from distal nerves of DRG neurons as a ‘glove and stocking’ sensory loss (Addington & Freimer, 2016; Argyriou et al., 2008; Mcwhinney et al., 2009). However, the majority of experimental studies in CIPN have primarily analyzed the effect of platinum drugs on cell bodies of DRG neurons and have not investigated the direct effects of platinum drugs on distal nerve fibres (Cata et al., 2006; Wang et al., 2000). EVs have been used as therapies for pain (Ren et al., 2019; Shiue et al., 2019) and diabetic peripheral neuropathy (Fan et al., 2020; Jia et al., 2018; Wang et al., 2020), as well as for cancer (Kalluri, 2016; Pitt et al., 2016); however, investigations of the effect of EVs on CIPN, in particular in tumour bearing animals, is limited. Our in vitro data showed that axonal application of oxaliplatin directly suppressed axonal growth, whereas CEC‐sEVs overcame this oxaliplatin side effect, which is consistent with emerging data showing that EVs internalized by axons can change axonal function (Jia et al., 2018; Zappulli et al., 2016; Zhang et al., 2017). In ovarian cancer cells, CEC‐sEVs enhanced the effect of oxaliplatin on cancer cell viability and invasion. Importantly, a therapeutic effect of CEC‐sEVs in combination with oxaliplatin was observed in tumour bearing mice. A dose of 3 × 10^11^ particle/injection used in the present study is within a dose range of sEVs from published studies (4 × 10^10^ to 4 × 10^11^ particles/injection) in rodent and primate models of diabetic peripheral neuropathy and cortical injury, respectively (Go et al., 2020; Wang et al., 2020; Williams et al., 2019; Xin et al., 2017). Thus, the present study, for the first time, demonstrates that the CEC‐sEVs are effective in ameliorating oxaliplatin‐induced CIPN, and enhance the effects of oxaliplatin on suppressing tumour growth, which could potentially translate into clinical application.

EVs can transfer their cargo biological materials including proteins and miRNAs to recipient cells, consequently leading to change recipient cell function. For example, cargo miR‐21, ‐27a and ‐146a of Schwann cells derived sEVs affect their target genes, PTEN, RhoA and Sema6A, respectively, in recipient DRG neurons, resulting in improvements neurological outcomes in animal models of diabetic peripheral neuropathy (Wang et al., 2020). In addition, cargo let‐7a, miR‐17, ‐23a and ‐125b of MSC‐sEVs suppressed their target genes toll‐like receptor 4 (TLR4) in DRG neurons that ameliorated diabetic peripheral neuropathy in the mice (Fan et al., 2020). The present study showed that the combination treatment resulted in the upregulation of a set of miRNAs (miR‐15b, ‐214 and ‐125b) in sciatic nerve tissue and cancer cells, which were inversely associated with protein levels of TRPV1 and Sarm1 in the nerve tissue, and GRASP55 and β‐catenin proteins in ovarian tumour, respectively. TRPV1 and Sarm1 are known to mediate axonal functions and are involved in CIPN (Geisler et al., 2016; Hara et al., 2013; Hohmann et al., 2017; Szallasi et al., 2007), while augmentation of GRASP55 and β‐catenin induces chemoresistance (Kraya et al., 2015; Nguyen et al., 2019; Tan et al., 2017; Teeuwssen & Fodde, 2019). The changed miRNAs and proteins formed distinct networks in sciatic nerve and cancer cells, which mediate nerve damage and tumour progression, respectively. Our finding is consistent with published data that a single miRNA can target several mRNAs (Baek et al., 2008; Bracken et al., 2016; Cortez‐Dias et al., 2016; Pillai, 2005) and the interaction of exosome cargo, in particular miRNAs, with recipient cells is cell type dependent (Fan et al., 2020; Jia et al., 2018; Mathieu et al., 2019; Tkach & Théry, 2016; Wang et al., 2020; Zhang et al., 2015). Ultrastructural data that CEC‐sEVs intravenously administered reached sciatic nerve fibres and tumour cells provide additional support that CEC‐sEVs can interact with sciatic nerve and cancer cells. Importantly, our in vitro data showed that reduction of Dicer‐related miRNAs within CEC‐sEVs abolished the effect of CEC‐sEVs on oxaliplatin‐induced axonal growth and on enhancement of the anti‐cancer effect of oxaliplatin. Specifically, cargo miR‐214 appears critical to the therapeutic effect of CEC‐sEVs by acting on its target genes in DRG neurons and OC cells. As predicted, the other two miR‐15b and ‐125b also likely play roles, although we did not investigate them. We and others recently demonstrated in addition to transferring their cargo miRNAs, internalized sEVs selectively trigger upregulation of miRNAs that consequently result in reduction of their target genes in recipient cells (Au Yeung et al., 2016; Baroni et al., 2016; Zhang et al., 2017, 2020). However, additional in vivo experiments to examine the causative effect of CEC‐sEVs carrying reduced or elevated individual miRNAs are warranted. Collectively, the present study suggests that CEC‐sEVs cargo miRNAs likely contribute the therapeutic effect of the combination of CEC‐sEVs with oxaliplatin on CIPN, although the contribution of cargo proteins cannot be excluded.

In summary, the present study indicates that CEC‐sEVs ameliorate oxaliplatin‐induced peripheral neuropathy and sensitize the anti‐tumour effect of oxaliplatin in the mouse bearing ovarian tumour. These data suggest that CEC‐sEVs have potential applications in platinum‐drug related cancer treatments.

## 4 METHODS AND MATERIALS

All animal experiments were carried out in accordance with the NIH Guide for the Care and Use of Laboratory Animals and approved by the Institutional Animal Care and Use Committee of Henry Ford Hospital.

### 4.1 Isolation and characterization of sEVs

Small EVs (CEC‐sEVs) were isolated from cultured primary human CECs (ACBRI376, Cell Systems, Figure 1a) according to our published protocol (Zhang et al., 2017). Briefly, CECs were cultured and subsequently passaged using Complete Classic Medium (4Z0‐500, Cell Systems). When the cell confluence reached to 60%–80%, the medium was replaced with complete serum‐free medium (SF‐4Z0‐500, Cell Systems) and cultured for an additional 48 h, which is a chemically defined complete medium that does not contain serum‐derived EVs (Niego & Medcalf, 2013; Paradis et al., 2016) and is suitable for sEV collection (Figure S8). The supernatant of the CEC medium was then collected and filtered through a 0.22 μm filter (Millipore, CA, USA) to remove all dead cells and large debris. A 10,000×*g* centrifugation for 30 min was performed to further remove small debris. After an ultracentrifugation at 100,000×*g* (Optima XE‐100 Ultracentrifuge, Beckman Coulter) for 2 h, the precipitation was re‐suspended in phosphate‐buffered saline (PBS) and preserved at 4℃ in refrigerator. The concentration and size distribution of CEC‐sEVs were determined by NanoSight NS300 (Malvern, UK), according to our published protocol (Wang et al., 2020). To further characterize sEVs, the ultrastructural morphology and sEV markers were characterized by transmission electron microscopy (TEM, JEOL JEM 1400) (Wang et al., 2020; Zhang et al., 2017) and Western blot (Xin et al., 2012), respectively. The following primary antibodies were used: mouse monoclonal anti‐Alix (1:500; 2171, Cell Signaling), rabbit polyclonal anti‐CD63 (1:500; ab34045, Abcam), rabbit polyclonal anti‐CD31 (1:500, MAB1393, Anti‐PECAM‐1, EMD Millipore), rabbit polyclonal anti‐ZO‐1 (1:500; 61–7300, Thermo Fisher Scientific), and rabbit monoclonal anti‐Calnexin (1:500, 699401, Biolegend).

### 4.2 Adult DRG neuron culture in microfluidic devices

Primary DRG neurons were isolated from female 6–8 week old C57BL/6J mice (000664, Jackson Laboratory), according to published protocols (Fornaro et al., 2018; Sleigh et al., 2016). Briefly, the sciatic nerve originating lumbar DRGs (L4 to L6) were extracted and digested in 1.25 mg/ml collagenase IV (07427, StemCell) for 45 min and then with 0.025% trypsin (diluted from 0.05% trypsin, 25300, ThermoFisher) for 30 min. The DRG neuron suspension was then obtained by mechanically dissociating DRGs via a glass pipette. The cell suspension was passed through a 40 μm cell strainer and counted to obtain a concentration of 1×10^6^cells/ml.

To separate axons from neuronal soma and examine the effects of oxaliplatin/CEC‐sEVs on axons of DRG neurons, a microfluidic device (Standard Neuron Device, Cat# SND150, Xona Microfluidics, Temecula, CA) was employed (Figure 1e) (Jia et al., 2018; Wang et al., 2020; Zhang et al., 2017). Briefly, sterilized devices were affixed to poly‐D‐lysine (PDL) (0.5 mg/ml, Sigma‐Aldrich, CA) and laminin (0.05 mg/ml, 23017015, ThermoFisher Scientific) ‐coated dishes (35 mm, Corning). The primary DRG neurons were plated at a density of 2×10^4^cells/chamber in Dulbecco's Modified Eagle Medium: Nutrient Mixture F‐12 (DMEM/F12) with 5% Fetal Bovine Serum (FBS) for 24 h. After that, cell culture was incubated with the addition of neurobasal growth medium, 2% B‐27, 2 mM GlutaMax, and 1% antibiotic‐antimycotic (Thermo Fisher Scientific, Waltham MA, USA), which was counted as the starting day in vitro (DIV). On DIV 3, one‐half of the medium was replaced with culture medium containing 20 μM 5‐fluorodeoxyuridine. The growth media was changed every other day thereafter. To examine the effect of CEC‐sEVs on axonal growth of DRG neurons, CEC‐sEVs at 3 × 10^7^ were concurrently treated with oxaliplatin at 0, 8.75, 17.5 and 35 nM into the axonal compartment of the microfluidic devices on DIV3 for 24 h. The growth cone extensions were then measured according to our published protocol (Zhang et al., 2013, 2015).

### 4.3 MTT assay

Briefly, human ovarian carcinoma SKOV3 and OVCAR3 cells at 1×10^3^ cells/well were seeded into 96‐well cell culture plate (Corning) 24 h before treatment. CEC‐sEVs at 3 × 10^7^, 3 × 10^8^ and 3 × 10^9^ alone, or in combination with oxaliplatin were added into designated wells. After 72 h, the medium was replaced with fresh growth medium containing 0.5 mg/ml thiazolyl blue tetrazolium bromide (MTT, Sigma‐Aldrich, M2128) and cultured for an additional 4 h. After carefully removing the growth medium with unreacted MTT, the purple formazan in 96‐well plates was then dissolved in 200 μL DMSO. The absorbance, indicating the amount of living cells, was then examined using a microplate reader (Molecular Devices SPECTRAMAX PLUS 384) at 450 nm and 570 nm. The inhibition rate was calculated by following formula: [(OD570 ‒ OD450) _treatment_ ‒ (OD570‐OD450) _control_] / (OD570 ‒ OD450) _control_ X 100%. The IC_50_ was calculated by linear regression to recall the concentration of OHP that causes 50% inhibition rate on ovarian cancer (OC) cells.

To assess whether the effects of sEVs on OC cells and axons of DRG neurons are specific, a set of controls were employed including: (1) Liposome mimics, which were generated via the thin‐film hydration technique (Ekanger et al., 2014); (2) EV‐depleted CEC growth medium, the supernatant of CEC growth medium after sEVs were pelleted by ultracentrifugation; and (3) NCM particles, which were collected from the medium placed in culture dishes but without CECs. NCM particles were dosed as the concentration of particle numbers measured by NTA. The sEV‐depleted medium at the equal volume of sEV solution was applied to the axonal compartment.

### 4.4 Wound healing assay

Briefly, OVCAR3 cells (2×10^5^ cells) were seeded into a 6‐well cell culture plate (Corning). After reaching to ∼80% confluence as a monolayer at 24 h culture, a straight scratch across the centre of the culture surface was introduced. The detached cells were then carefully washed away with PBS and replaced with fresh growth medium. Five phase contrast images along with each scratch were longitudinally then captured with Nikon eclipse Ti microscope under a 4x objective before and 12 h after treatment. The gap areas of each image were then measured by Image J. The data are presented by relative scratch areas as the rate of average gap area in the conditions of 12 h treatment versus before treatment (0 h).

### 4.5 Transwell migration assay

A Corning BioCoat Matrigel Invasion Chamber (Fisher Scientific, 08‐774‐122) was employed. Briefly, the transwell inserts in 24‐well plates were rehydrated in 37℃, 5% CO_2_ cell culture incubator for 2 h with serum‐free RPMI‐1640 medium. OVCAR3 cells in serum‐free RPMI‐1640 medium were then seeded into the inserts at 2.5×10^4^ cell/0.5 ml. Complete medium with 10% FBS was then added into the wells of 24‐well plates (out of the insert) for 0.75 ml per well. CEC‐sEVs at 3 × 10^8^ alone or in combination with oxaliplatin at 1/3 μM were added into the medium in transwell inserts. After 24 h culture, 1 μL (0.5 μg) cell tracker (CellTracker Red CMTPX, ThermoFisher, C34552) was added into the medium in 24‐well plates for 15 min, which followed with the fixation by 4% paraformaldehyde (PFA). The non‐invading cells were then removed from the inner surface of the transwell insert by cotton swab. The bottom surfaces of transwell insert were imaged with a fluorescent microscope under a 10x objective. Ten images per insert were acquired and the data are presented as the average cell number of each insert.

### 4.6 CIPN mouse model on OC bearing nude mouse

Female nude mice bearing OC tumours were employed. Briefly, female BALB/c nude mice at age of 6 weeks (Charles River, Wilmington, MA, USA) were used. For the subcutaneous (s.c.) xenograft model, 5 × 10^6^ SKOV3/luc cells were injected into the dorsum of nude mice. After approximately 4‐week growth, the tumours were excised and chopped into 3 × 3 × 3 mm^3^ pieces and s.c. implanted into the dorsum in new cohorts of female BALB/c nude mice 1 week before treatments. For the intraperitoneal (i.p.) xenograft model, 5 × 10^6^ OVCAR3/luc cells were injected i.p. 2 weeks before treatments. To induce CIPN on these mice, oxaliplatin (3.0 mg/kg, i.p.) was administered daily for two rounds of five consecutive days per week at week 1 and week 3, with 1‐week rest at week 2, which mimics the clinical protocol (Figure 3a). These OC tumour bearing mice (*n* = 7/group) were randomly treated with: (1) oxaliplatin in combination with CEC‐sEVs, (2) oxaliplatin alone, (3) CEC‐sEVs alone and 4) PBS. A dose of CEC‐sEVs (3 × 10^11^ particles) selected based on preclinical studies in rodent, swine and primate (Go et al., 2020; Wang et al., 2020; Williams et al., 2019; Xin et al., 2017) was administered via a tail vein three times per week for six consecutive weeks starting from the same day with oxaliplatin treatment. The CIPN related symptoms of cold hyperalgesia and tactile allodynia sensory were measured by cold plate and Von Frey assays, respectively. Nerve conduction velocities in the sciatic nerve were measured bilaterally by electrophysiology assay. Tumour size was longitudinally and non‐invasively imaged weekly by bioluminescence imaging (BLI) machine (IVIS Spectrum, Caliper Life Sciences). The body weights were recorded weekly as the indication of toxicity induced by oxaliplatin (Sprowl et al., 2013). All mice were sacrificed 8 weeks after xenograft implantation (Figure 3a).

### 4.7 Cold plate assay

Briefly, mice were place into Plexiglas cylinder on the metal plate of Cold plate Analgesia Meter (IITC Life Science, Woodland Hills, CA) for 2 min. The temperature of metal plate were set to ‐4±0.2°C with the consideration of no tissue damage and the lowest variability (Ta et al., 2009). The times of quick paw lifts and jumps that demonstrate cold hyperalgesia were counted by two independent investigators according to published protocols (Biessels et al., 2014; Pande et al., 2011; Sullivan et al., 2007; Ta et al., 2009; Wang et al., 2015).

### 4.8 Von Frey assay

Briefly, mice were placed in individual Plexiglas cubicles on a wire mesh platform, and allowed to acclimate for approximately 30 min. The Von Frey filaments (Stoelting, USA) were employed to stimulate paw withdrawal. A series of filaments with bending force that ranged from 0.4 to 6.0 g were applied to the plantar surface of the left hind paw with pressure causing the filament to buckle. A paw withdrawal in response to each stimulus was recorded and a 50% paw withdrawal threshold was calculated according to published protocols (Chaplan et al., 1994; Wang et al., 2020).

### 4.9 Electrophysiology assay

Briefly, mice were anesthetized and placed on a water heating pad at 37±0.5°C. The stimulating electrodes were placed at the knee and sciatic notch. Triggered single square wave current pulses were delivered using an isolated pulse stimulator (Model 2100, A‐M Systems, USA). The simultaneous electromyographies were recorded by two sterilized electrodes placed in the dorsum of the foot with a Grass Amplifier (Model P5, Grass Instruments, USA). MCV and SCV in the sciatic nerve were measured according to published protocols (Ii et al., 2005; Wang et al., 2015, 2020).

### 4.10 CEC‐sEV labelling and tracking

In order to trace distribution of intravenously administered CEC‐sEVs in tumour and sciatic nerves of tumour bearing mice, we generated CEC‐sEVs carrying CD63‐GFP (GFP‐sEVs) according to our published protocols (Wang et al., 2020). Briefly, CECs were transfected with a plasmid carrying pEGFP‐CD63 vector (CD63‐pEGFP C2, a gift from Paul Luzio, Addgene plasmid # 62964) by means of electroporation (Nucleofector system, program U11, Lonza) (Zhang et al., 2013). CD63 is a membrane protein marker of exosomes (Kowal et al., 2016). The presence of GFP proteins in sEVs (GFP‐sEVs) was examined by means of Western blot.

GFP‐sEVs were administrated i.v. into tumour bearing mice 2 h before sacrifice. The xenografts and sciatic nerve tissues were excised and fixed. The fixed tissues were then cut into 100 μm‐thick sections using a vibratome. ProLong Diamond Antifade Mountant with DAPI were applied to each section overnight to label the nucleus. The immunoreactive samples were imaged under a 63x objective by means of a laser‐scanning confocal microscope (Zeiss LSM 510 NLO, Carl Zeiss, Germany) (Zhang et al., 2009, 2013).

To further trace the internalization of CEC‐sEVs at the ultra‐structural level, immunogold staining with TEM analysis was performed according to our published protocols (Wang et al., 2020). Briefly, fixed tumour xenografts and sciatic nerve tissues were embedded and cut into ultrathin sections (80 nm) and loaded on nickel grids. Immunogold staining was performed on the grids with 2% anti‐rabbit monoclonal antibody against GFP (G10362, ThermoFisher) and 10 nm gold conjugated streptavidin (25269, EMS). The grids were imaged under the TEM (JEM‐1500Flash, JEOL).

### 4.11 Knockdown of Dicer and miR‐214 in CEC‐sEVs

To examine the cargo miRNAs are required for the effects of CEC‐sEVs, CEC‐sEVs with knockdown of Dicer (Dp‐CEC‐sEVs) and miR‐214 (Zipm214‐CEC‐sEVs) were isolated from CECs transfected with Dicer shRNA plasmid (sc‐40489‐SH, Santa Cruz) or lentiviral vector that carrying shRNA against miR‐214 (MZIP214‐PA‐1, SystemBio) by electroporation (Zhang et al., 2020), respectively, whereas the control sEVs, scra‐CEC‐sEVs, were isolated from CECs transfected with a vector expressing scramble shRNA (MZIP000‐PA‐1, SystemBio).

### 4.12 Immunohistochemistry and image quantification

To examine the effect of CEC‐sEVs and oxaliplatin on DRG neurons and IENF, immunofluorescent staining was performed on DRG (8 μm thick) and footpad tissue (20 μm thick) sections, respectively, according to our published protocols (Zhang et al., 1999, 2010, 2013, 2014). The following antibodies were employed: polyclonal anti NF200 (N4142, MilliporeSigma), CGRP (ab43873, abcam) and monoclonal anti‐PGP9.5 (ab8189, Abcam). Immunoreactive cells and nerve fibres were imaged using laser scanning confocal microscopy (LSCM) and were quantified according to our published protocols (Buller et al., 2010; Wang et al., 2014).

To identify the morphometric changes within sciatic nerves, toluidine blue staining was performed on semi‐thin transverse sections (2 μm thick) of sciatic nerve and imaged using a 100x oil immersion lens (Olympus). Myelin sheath thickness, myelinated fibre and axon diameter were measured using the MCID system, as described in our published protocols (Zhang et al., 1999, 2010, 2013, 2014). The myelin sheath and axons in ultra‐structural level were also analyzed by means of TEM.

### 4.13 MiRNA sequencing and quantitative real‐time reverse transcriptase‐polymerase chain reaction (qRT‐PCR)

To isolate total RNAs from CEC‐sEVs, tumour xenografts or sciatic nerve tissues, CEC‐sEVs or homogenized tissues collected from nude mice were lysed in Qiazol reagent, and total RNA was isolated using the miRNeasy Mini kit (Qiagen, Valencia, CA, USA), as reported (Zhang et al., 2013, 2015, 2016). MiRNA profiles were analyzed by means of miRNA sequencing, according to our published protocols (Liu et al., 2011, 2017). Briefly, the total RNAs were used to establish the miRNA sequencing library via the data from Agilent 2100 Bioanalyzer. Sequencing was carried out using the Illumina NextSeq 500. The expression level (Reads count) of miRNA was calculated using mirdeep2.

In order to verify the levels of candidate miRNAs identified in miRNA sequencing, qRT‐PCR was performed with TaqMan miRNA assay kit (ThermoFisher Scientific). Briefly, miRNAs were reversely transcribed with the miRNA Reverse Transcription reagent and amplified with Taqman PCR reagents, which were specifically designed for detecting mature miR‐15b‐5p sequence (UAGCAGCACAUCAUGGUUUACA), miR‐214‐3p sequence (ACAGCAGGCACAGACAGGCAGU) and miR‐125b‐5p sequence (UCCCUGAGACCCUAACUUGUGA). U6 snRNA (mature sequence: GTGCTCGCTTCGGCAGCACATATACTAAAATTGGAACGATACAGAGAAGATTAGCATGGCCCCTGCGCAAGGATGACACGCAAATTCGTGAAGCGTTCCATATTTT) was used as the internal control. In order to compare the group differences, relative expression levels were calculated by 2^−ΔΔCt^ method according to published protocol (Livak & Schmittgen, 2001).

### 4.14 Western blot analysis

Tumour xenografts and sciatic nerve tissues were homogenized in lysis buffer (RIPA, Sigma‐Aldrich) and continued to be lysed on ice for 30 min. The supernatant was then collected after centrifugation at 12,000 r.p.m, 4℃ for 15 min. The protein concentrations were analyzed with a bicinchoninic acid (BCA) protein assay kit (Pierce Biotechnology, Rockford, IL). Western blot was performed according to published protocols (Zhang et al., 2013, 2015). Briefly, equal amounts of total protein for each sample were loaded on 10% SDS‐polyacrylamide gels. After electrophoresis, the proteins were transferred to Polyvinylidene difluoride (PVDF) membrane by Trans‐Blot Turbo System (Bio‐Rad). After drying for 1 h on a clean filter paper and blocking in 0.2% I‐block solution (T2015, ThermoFisher Scientific), the membrane was incubated with primary antibody overnight at 4 ℃. The next day, horseradish peroxidase (HRP)‐conjugated secondary antibodies were used (1:2000) for 2 h at room temperature. Enhanced chemiluminescence development kit (Pierce Biotechnology) was employed to detect the bands by a chemiluminescence imaging system (FluroChem E, protein simple) and analyzed using quantitative software (AlphaView, protein simple). The following primary antibodies were used in this study: rabbit polyclonal anti SARM1 (1:1000, LS‐B13, LS Bio), rabbit polyclonal anti TRPV1 (1:1000, acc‐030, alomone), rabbit polyclonal anti NGF (1:1000, ab6198, abcam), rabbit polyclonal anti GRASP55 (1:1000, PA1‐076, ThermoFisher), rabbit polyclonal anti β‐catenin (1:1000, E2264, Spring Biosci.), and mouse monoclonal anti β‐actin (1:10000, ab6276, Abcam), mouse monoclonal anti CD31 (1:1000, M0823, Dako), rabbit polyclonal anti caspase‐3 (1:1000, 9662, Cell Signaling) and mouse monoclonal anti Alix (1:1000, 2171, Cell Signaling). The whole Western blots images are provided in Figure S9.

### 4.15 Statistical analysis

All statistical analysis was performed using the Statistical Package for the Social Sciences (SPSS, version 11.0; SPSS Inc, Chicago, IL, USA) and GraphPad Prism 8 (version 8.2.1, San Diego, CA, USA). One‐way ANOVA with Tukey's multiple comparisons test was used when comparing more than two groups. Student's *t* test was used when comparing two groups. Values presented are expressed as mean ± standard error. A *P*‐value < 0.05 was considered to be significant.

## CONFLICT OF INTEREST

No

## AUTHOR CONTRIBUTIONS

Yi Zhang and Zheng Gang Zhang designed the experiments; Yi Zhang, Chao Li, Yi Qin, Pasquale Cepparulo, Michael Millman, Amy Kemper, Alexandra Szalad, Xuerong Lu and Lei Wang performed experiments and analyzed data, Yi Zhang, Michael Chopp and Zheng Gang Zhang wrote the manuscript.

## Supporting information



Supporting Information

Supporting Information

## Data Availability

The data that support the findings of this study are available from the corresponding authors, upon reasonable request.
