# Supplementary material for: Small extracellular vesicles ameliorate peripheral neuropathy and enhance chemotherapy of oxaliplatin on ovarian cancer
Source: J Extracell Vesicles. 2021 Mar 4;10(5):e12073. doi: 10.1002/jev2.12073 (PMC7931803; doi:10.1002/jev2.12073)
Supplement: Supplementary file 1 — Supporting Information [file JEV2-10-e12073-s001.docx]

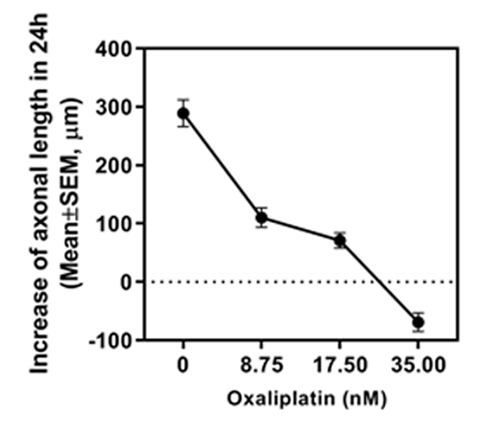


**Supplemental Figure 1**. **Oxaliplatin suppresses the growth of DRG axons.** Quantitative data show changes of axonal length during a 24 hour period after oxaliplatin at various concentrations was applied into the axonal compartment of microfluidic device SND150. A minus value means the decrease of axonal length. Error bars indicate the standard error of the mean (SEM). All the data were from 3 independent experiments.


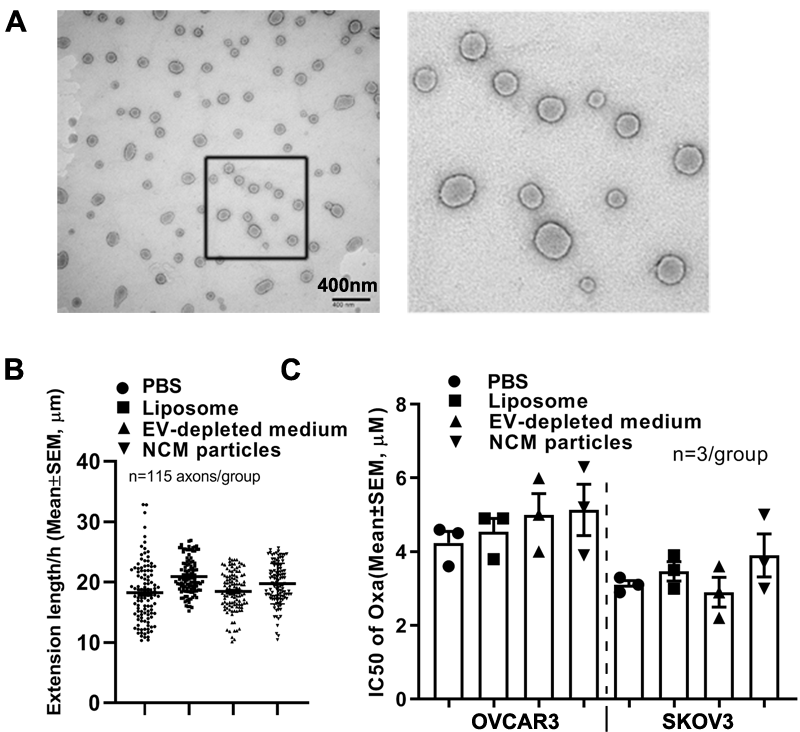


**Supplemental Figure 2**. **The effects of control particles/medium on axonal growth of DRG neurons and OC cells, respectively.** A representative TEM image with high magnification of the boxed area (A) shows the ultrastructural morphologies of particles isolated from non-conditioned medium (NCM). Quantitative data (B) show the effect of axonal applications of liposomes, EV-depleted medium, and NCM particles, respectively, on growth cone extension of DRG neurons during a 24 hour period. Quantitative MTT data (C) show that the applications of liposomes, EV-depleted medium, and NCM particles, respectively, did not alter the IC_50_ of oxaliplatin on cell viability of OVCAR3 and SKOV3 cells. One-way ANOVA with Tukey’s multiple comparisons test was used. All the data were from 3 independent experiments.


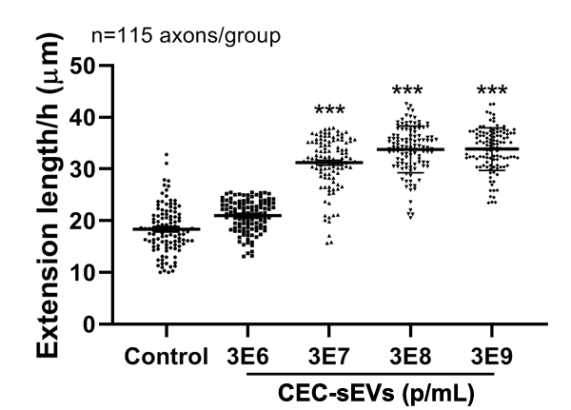


**Supplemental Figure 3**. **A dose-response of axonal treatment of CEC-sEVs on the extension of growth cone of DRG neurons**. One-way ANOVA with Tukey’s multiple comparisons test was used. *** p<0.001 vs control. Error bars indicate the SEM.


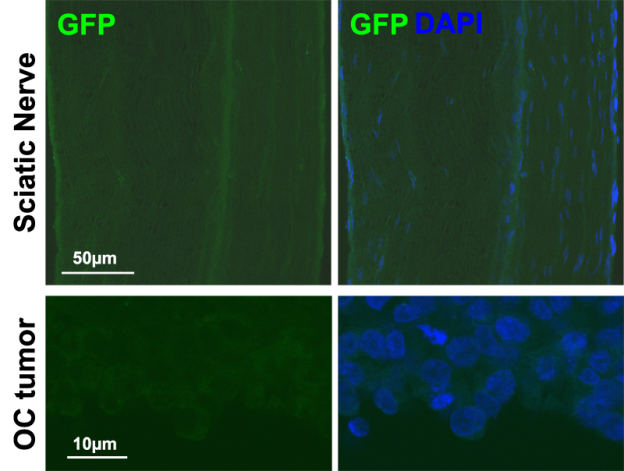


**Supplemental Figure 4**. **Nano particles isolated from NCM did not exhibit fluorescent signals.** Representative confocal microscopic images show that there were no green fluorescent signals in the sciatic nerve and OC tumor of mice at 4h after treatment with NCM particles.


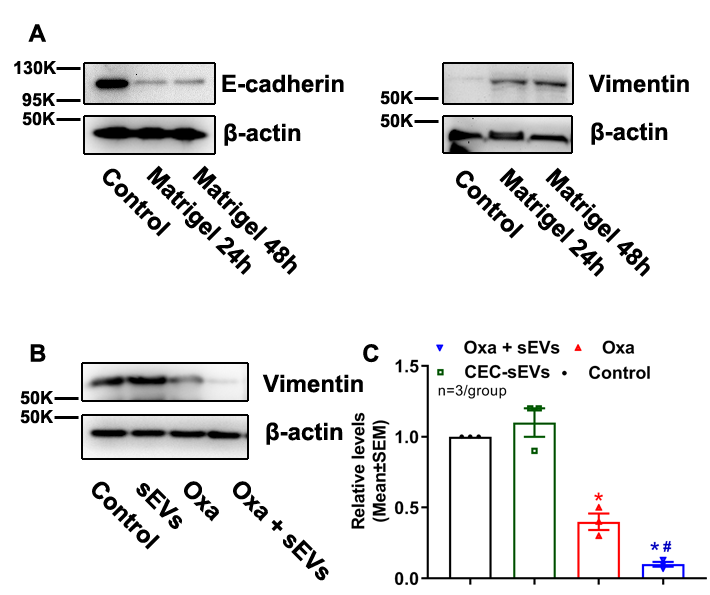


**Supplemental Figure 5**. **CEC-sEVs augment oxaliplatin-induced phenotype changes of OVCAR3 cells**. Representative Western blot images (A) show the levels of epithelial maker, E-cadherin, and mesenchymal marker, vimentin, in OVCAR3 cells after 24h and 48h culture in Matrigel, respectively. Representative Western blot images (B) and the quantitative data (C) show the treatment of oxaliplatin (1/3µM) significantly decreased the levels of vimentin in OVCAR3 after 48h culture in Matrigel, whereas the combination of 3x10^8^ p/mL CEC-sEVs and oxaliplatin further decreased the levels of vimentin. One-way ANOVA with Tukey’s multiple comparisons test was used. * p<0.05 vs control. #, p<0.05 vs Oxa. Error bars indicate the SEM. All the data were from 3 independent experiments.


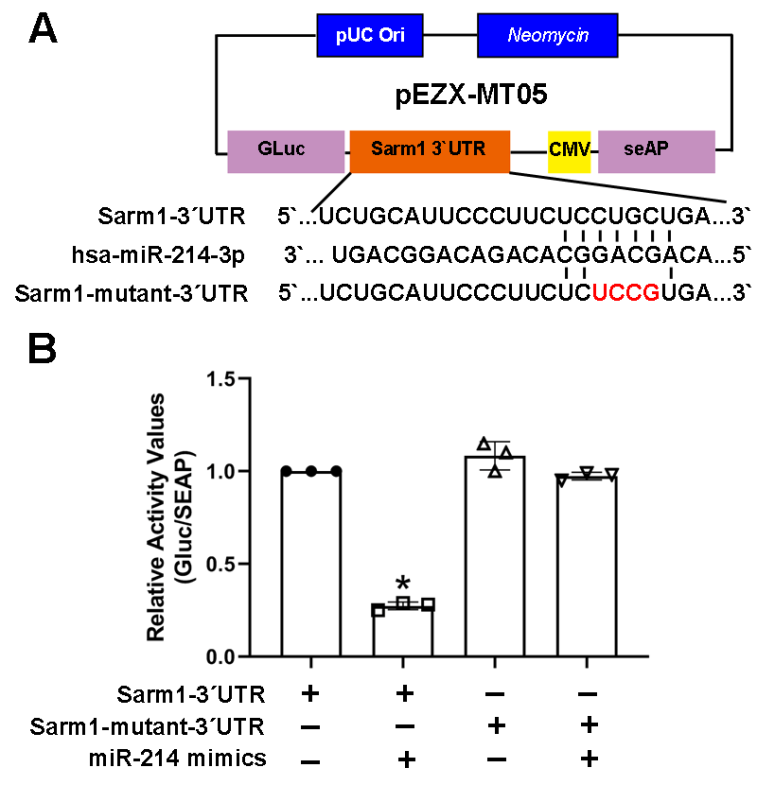


**Supplemental Figure 6**. **miR-214-3p directly binds to 3`UTR of Sarm1 gene.** A schematic shows a dual-luciferase reporter vector (pEZX-MT05, GeneCopoeia) carrying 3`UTR sequences of Sarm1(Sarm1-3`UTR) and the target region of consequential pairing by has-miR-214-3p. Sequences with red are mutant binding sites on Sarm1-mutant-3`UTR. HEK293T cells were transfected with these reporter plasmids and hsa-miR-214-3p mimics (PM12124, ThermalFisher Scientific). A Secrete-Pair™ Dual Luminescence Assay System was used to measure the luciferase activities of Gluc and an internal control SEAP. The quantitative data in B show that HEK293T cells co-transfected with Sarm1-3`UTR and miR-214 mimics show significantly reduction (>70%) of luciferase activities compared with cells transfected with Sarm1-3`UTR alone. However, mutation of miR-214 binding site in 3`UTR of Sarm1 abolished reduction of luminescence activity by miR-214 mimics. These data indicate that miR-214 regulates expression of Sarm1 by directly binding to 3`UTR of the Sarm1 gene.


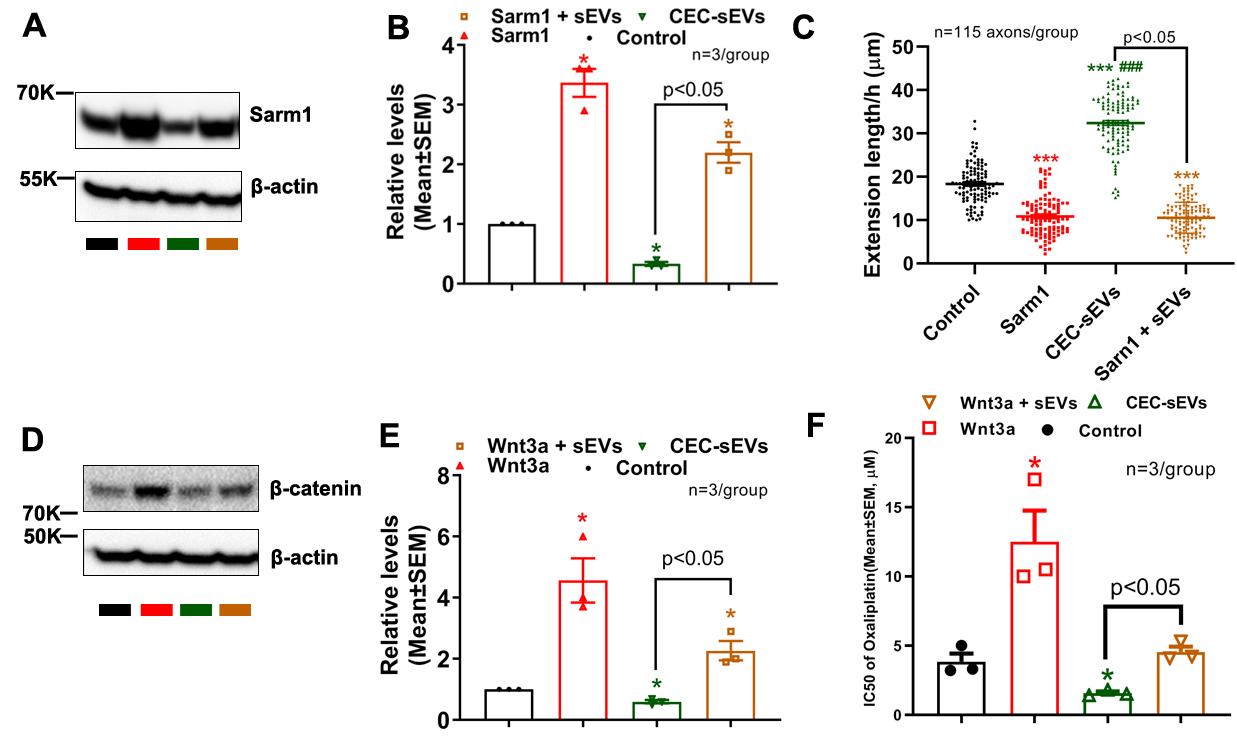


**Supplemental Figure 7**. **Overexpression of Sarm1 in DRGs or activation of the Wnt/β-catenin pathway in OVCAR3 cells attenuates the effects of CEC-sEVs on axons and OC cells, respectively, in the presence of oxaliplatin.** Representative (A) and quantitative Western blot data (B) show that compared with the control, Sarm 1 protein was significantly increased in DRG neurons transfected with a plasmid carrying Sarm1, which led to significant decreases in growth cone extension (C, red). Treatment of axons of Sarm1 expressed DRG neurons with CEC-sEVs did not significantly increase growth cone extension compared to DRG neurons with Sam1 overexpression (C). Treatment of OVCAR3 cells with Wnt3a (100ng/mL) for 24h significantly increased the levels of β-catenin (D, E) and increased the oxaliplatin IC50 on OVCAR3 cells (F, red). Compared with CEC-sEV treatment of OVCAR3 cells without Wnt3a, the treatment of Wnt3a treated OVCAR3 cells with CEC-sEVs significantly increased β-catenin (D, E) and oxaliplatin IC50 (F). A plasmid to overexpress Sarm1([114](#_ENREF_114)) (pGW1-Myc-Sarm1 was a gift from Yi-Ping Hsueh (Addgene plasmid # 50707 ; http://n2t.net/addgene:50707 ; RRID:Addgene_50707) was employed to overexpression Sarm1 in DRG neurons by electroporation with a protocol that we previously used ([18](#_ENREF_18), [115](#_ENREF_115)). N indicates the number of replications. One-way ANOVA with Tukey’s multiple comparisons test was used. * p<0.05, *** p<0.001 vs control. ### p<0.001 vs Sarm1 in A. Error bars indicate the SEM. All the data were from 3 independent experiments.


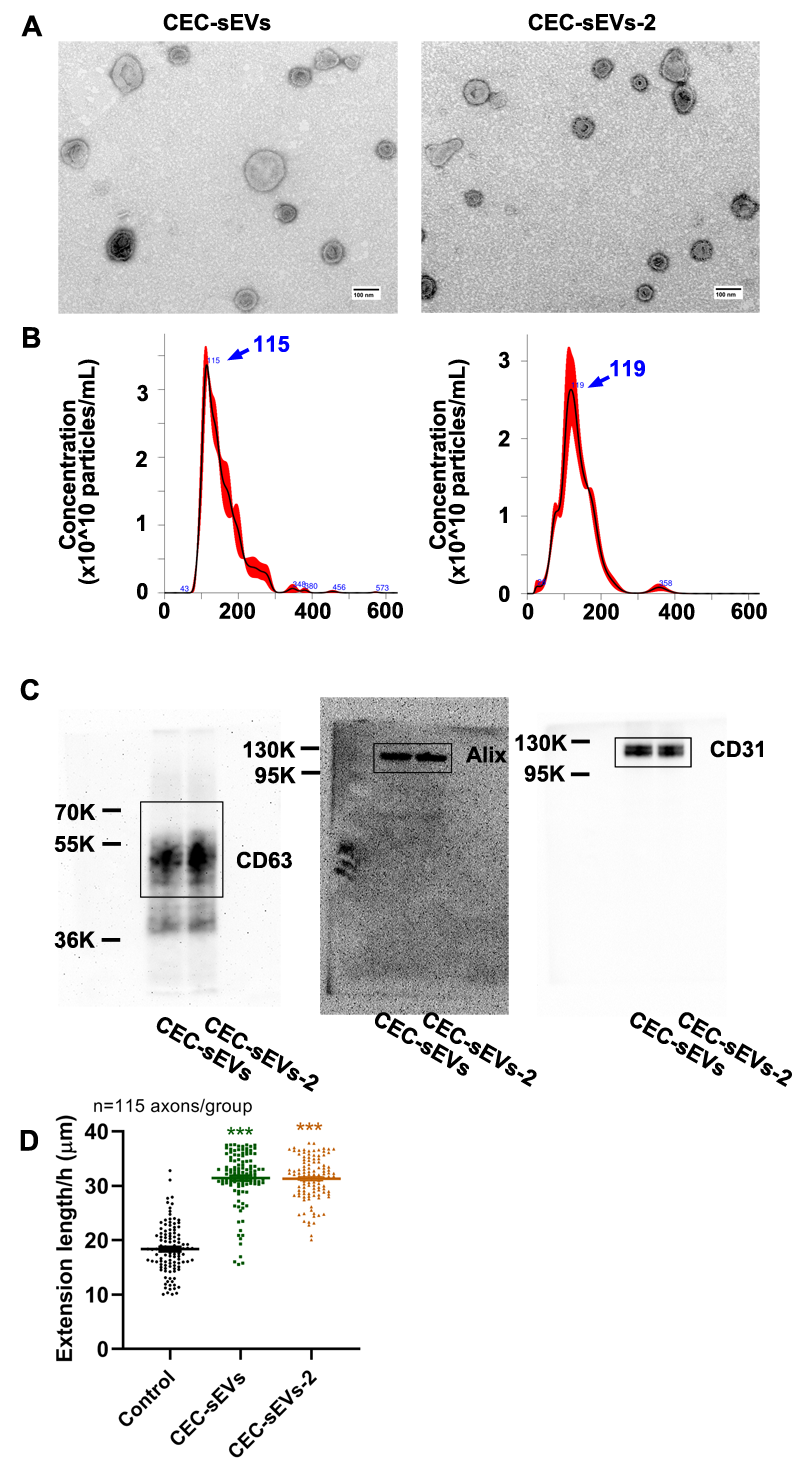


**Supplemental Figure 8**. **The comparison of sEVs isolated from the medium collected from CECs cultured with sEV-depleted serum (CEC-sEVs-2) with sEVs isolated from medium collected from CECs cultured with SF-4O-500 (CEC-sEVs).** There were no significant differences of sEV characterizations by means of TEM (A), NTA (B) and Western blots (C), respectively, between CEC-sEVs-2 and CEC-sEVs. Compared to the control group, both sEVs significantly increased axonal growth of DRG neurons (D). K = the molecular weight Kda. One-way ANOVA with Tukey’s multiple comparisons test was used. * p<0.05 vs control. Error bars indicate the SEM. All the data were from 3 independent experiments.


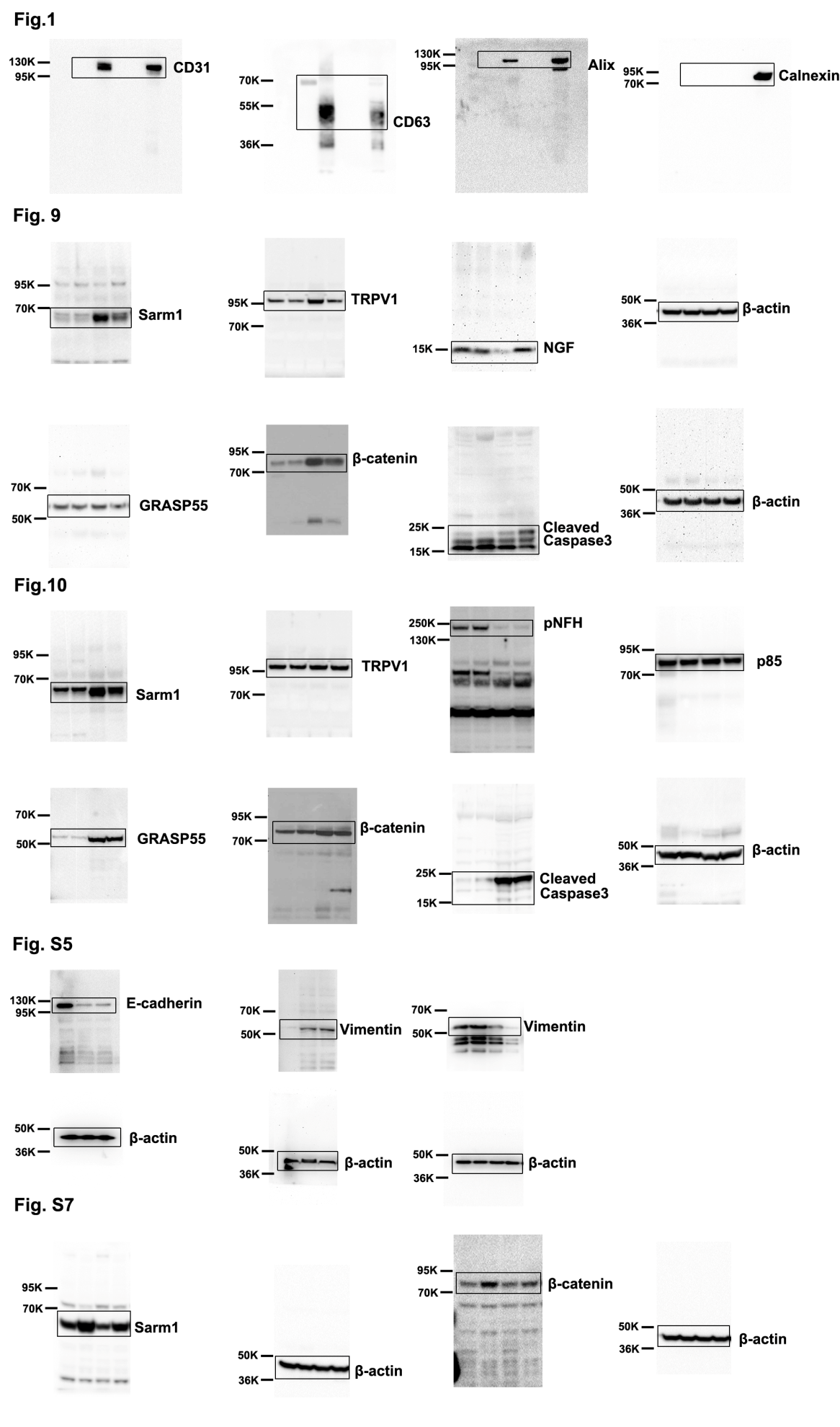


**Supplemental Figure 9**. Whole Western blots images of Figures 1, 9, 10 and supplemental Fig 5 and 6**.**

**Supplemental Table 1.** Comet Assay of OVCAR3 cells**.**

|  | **Average tail moments (TMs)** | | | | **oxa-DNA crosslinks** | |
| --- | --- | --- | --- | --- | --- | --- |
| Tests | Control | H2O2 | Oxaliplatin | Oxa + sEVs | Oxaliplatin | Oxa + sEVs |
| 1 | 11.3 | 23.7 | 3.3 | 4.3 | 165 | 156 |
| 2 | 10.5 | 25.3 | 2.2 | 3.3 | 156 | 149 |
| 3 | 11.6 | 22 | 3.4 | 4.1 | 179 | 172 |
| Total |  | | | | 166±7 | 159±7 |
|  | | | | | p>0.05 | |

The extents of oxaliplatin-DNA interstand crosslinks in OVCAR3 were analyzed by an alkaline comet assay. OVCAR3 cells in the 96-well plate were treated with oxaliplatin at 25μM for 12h (Oxa) or with oxaliplatin in combination with CEC-sEVs (3x10^8^ p/mL) for 12h (Oxa + sEVs). After that, cells were further treated with H2O2 (100 µM) for 15min to break DNA strands. The level of oxa-DNA crosslinks was calculated by formula: [1 − (TM_t_ − TM_ctl_)/(TM_H2O2_ − TM_ctl_)] × 100. TM_t_ indicates the treatment groups, TM_ctl_ indicates the non-treated control group, TM_H2O2_ indicates the H2O2 treated group.
